# Crystal structures of two formamidinium hexa­fluorido­phosphate salts, one with batch-dependent disorder

**DOI:** 10.1107/S2056989023010848

**Published:** 2024-01-01

**Authors:** Michelle C. Neary, Peter W. R. Corfield, Sean R. Parkin, Shahrokh Saba

**Affiliations:** a Department of Chemistry, Hunter College, The City University of New York, New York, 10065 NY, USA; b Department of Chemistry, Fordham University, 441 East Fordham Road, Bronx, NY 10458, USA; c Department of Chemistry, University of Kentucky, Lexington, KY, 40506-0055, USA; Vienna University of Technology, Austria

**Keywords:** crystal structure, formamidinium ion, nitro­gen heterocycles, hexa­fluorido­phosphate, hydrogen bonding, disorder

## Abstract

Syntheses of the acyclic amidinium salts, morpholino­formamidinium hexa­fluorido­phosphate [OC_4_H_8_N—CH=NH_2_]PF_6_ or C_5_H_11_N_2_O^+^·PF_6_^−^, **1**, and pyrrolidinoformamidinium hexa­fluorido­phosphate [C_4_H_8_N—CH= NH_2_]PF_6_ or C_5_H_11_N_2_^+^·PF_6_^−^, **2**, were carried out by heating either morpholine or pyrrolidine with triethyl orthoformate and ammonium hexa­fluorido­phosphate. Crystals of **1** obtained directly from the reaction mixture contain one cation and one anion in the asymmetric unit. The structure involves cations linked in chains parallel to the *b* axis by N—H⋯O hydrogen bonds in space group *Pbca*, with glide-related chains pointing in opposite directions. Crystals of **1** obtained by recrystallization from ethanol, however, showed a similar unit cell and the same basic structure, but unexpectedly, there was positional disorder [occupancy ratio 0.639 (4):0.361 (4)] in one of the cation chains, which lowered the crystal symmetry to the non-centrosymmetric space group *Pca2_1_*, with two cations and anions in the asymmetric unit. In the pyrrolidino compound, **2**, cations and anions are ordered and are stacked separately, with zigzag N—H⋯F hydrogen-bonding between stacks, forming ribbons parallel to (101), extended along the *b*-axis direction. Slight differences in the delocalized C=N distances between the two cations may reflect the inductive effect of the oxygen atom in the morpholino compound.

## Abstract 2

The crystal structures of two nitro­gen heterocycle hexa­fluorido­phosphate salts with terminal animinium groups are described. Unexpectedly, there is cation disorder in crystals of the morpholino­formamidinium compound obtained from ethanol recrystallization, which is not found in the original crystals.

## 1. Chemical context

The stability of N-heterocyclic carbenes and their applications in organic syntheses and in transition-metal catalysis has led in the past to intense inter­est in the syntheses of their precursors: cationic N-heterocyclic amidinium salts (Benhamou *et al.*, 2011 ▸). Previously, one of us reported a simple and efficient one-pot procedure for the preparation of cyclic amidinium salts by exchange reactions of various orthoesters with primary and secondary α,ω-di­amines in the presence of ammonium tetra­fluorido­borate or ammonium hexa­fluorido­phosphate (Saba *et al.*, 1991 ▸). This approach has been widely used for the preparation of cyclic amidinium salts in which the nitro­gen-flanked carbon atom bears a hydrogen atom and the nitro­gen atoms bear bulky substituents (for example: Funk *et al.*, 2006 ▸; Scarborough *et al.*, 2005 ▸). The use of orthoesters was then extended for the preparation of various acyclic amidinium hexa­fluorido­phosphates as potential carbene precursors (Saba *et al.*, 2005 ▸). We present here the first single-crystal structure determinations of these types of amidinium salts with N-heterocycles, *viz.* morpholino­formamidinium hexa­fluorido­phosphate, **1**, and pyrrolidinoformamidinium hexa­fluorido­phosphate, **2**. 
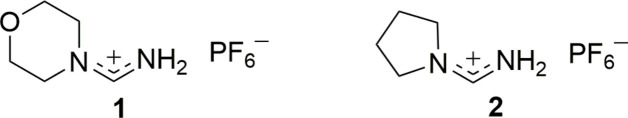


## 2. Structural commentary

**Figure 1:**
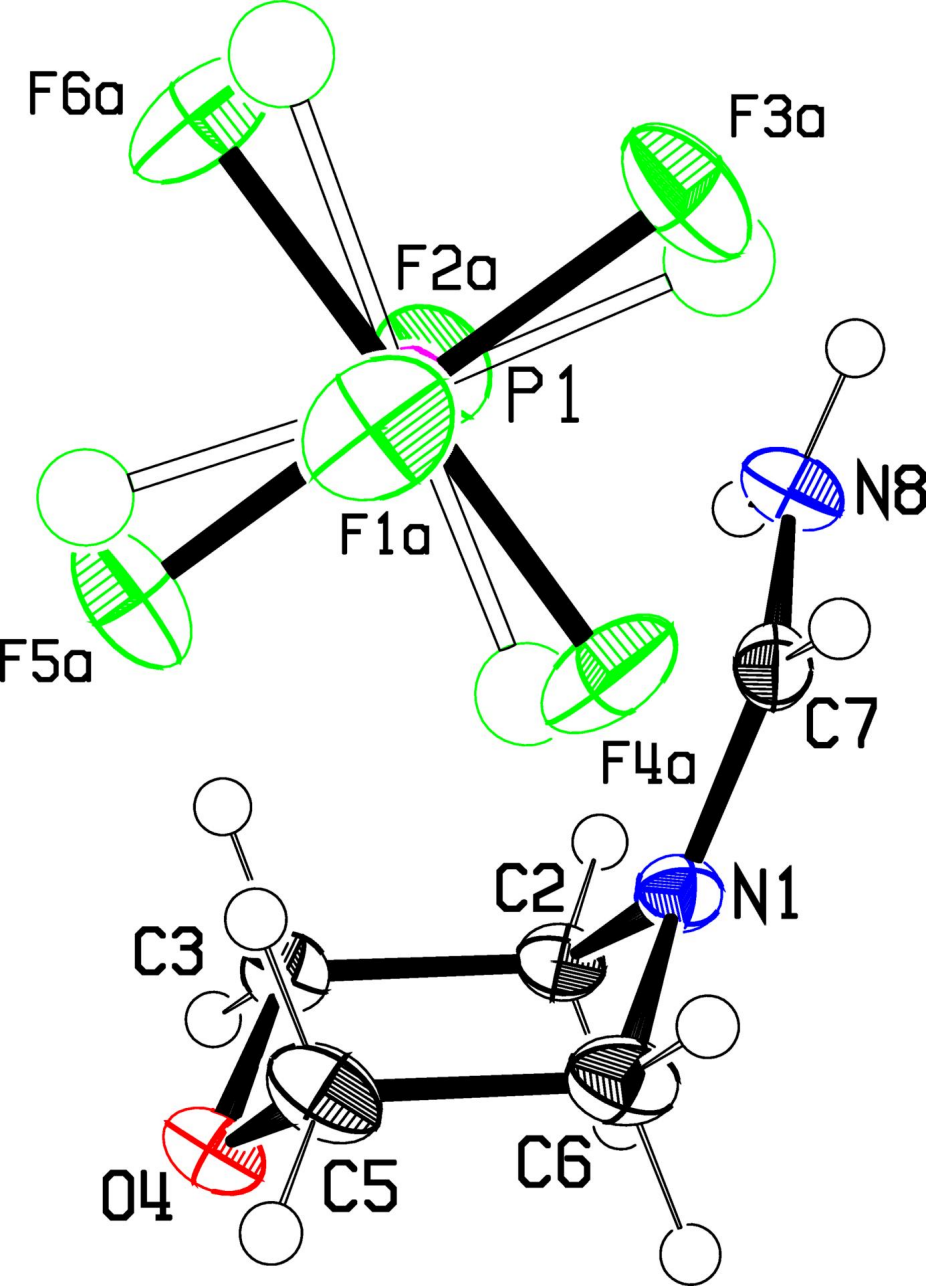
The asymmetric unit of **1**, showing the atom numbering. Displacement ellipsoids are drawn at the 50% probability level, while displacement parameters for the H atoms are arbitrary. The minor disordered PF_6_^−^ component is shown fa­inter. O atoms are colored red, N blue, C and H black, P magenta and F green.

The crystal structure of **1** was determined from crystals obtained directly from the original preparation described in the *Synthesis* section, and also from crystals obtained by recrystallization from ethanol, **1**(recryst). The structures of the mol­ecular moieties with atom numbering are shown in Figs. 1 ▸ and 2 ▸.

In **1**, the length of the delocalized C=N bond of 1.3016 (15) Å for the terminal C7—N8 bond is close to but slightly less than the value of 1.3142 (13) Å found for the bond adjacent to the ring, N1—C7; the angle at the methine C7 atom is 125.98 (10)°. The formamidinium group N1—C7H—N8H_2_^+^ is very close to planar with a root-mean-square (r.m.s.) deviation of the six atoms from the plane of 0.0050 Å. This group is not coplanar with the C2–N1–C6 plane of the morpholine group but is tilted by 13.4 (3)° from that plane. The morpholine moiety has the usual chair configuration, with the four atoms C2, C3, C5, C6 rigidly coplanar with an r.m.s. deviation of 0.0048 Å, and the O and N ends tilted by 52.6 (1) and 54.4 (1)°, respectively, from this plane.

Part of the sample was recrystallized from ethanol, in order to obtain larger crystals. Data from these crystals, **1**(recryst), indicated essentially the same unit cell but with intensities that did not exactly match those obtained for the original crystal. This intensity difference was shown to be due to disorder in one of the hydrogen-bonded cation chains, discussed in the next section, which lowered the symmetry to space group *Pca*2_1_ where two independent cations and anions are present. The shape of the cations are the same as found for the original crystal, albeit with somewhat less precision because of the disorder. To our knowledge, such a batch-dependent disorder is not often reported. Solvent-dependent disorder for some cobalt and zinc complexes is discussed in McCormick *et al.* (2018 ▸), but in that case there is solvate actually present in the crystal structures.

**Figure 3:**
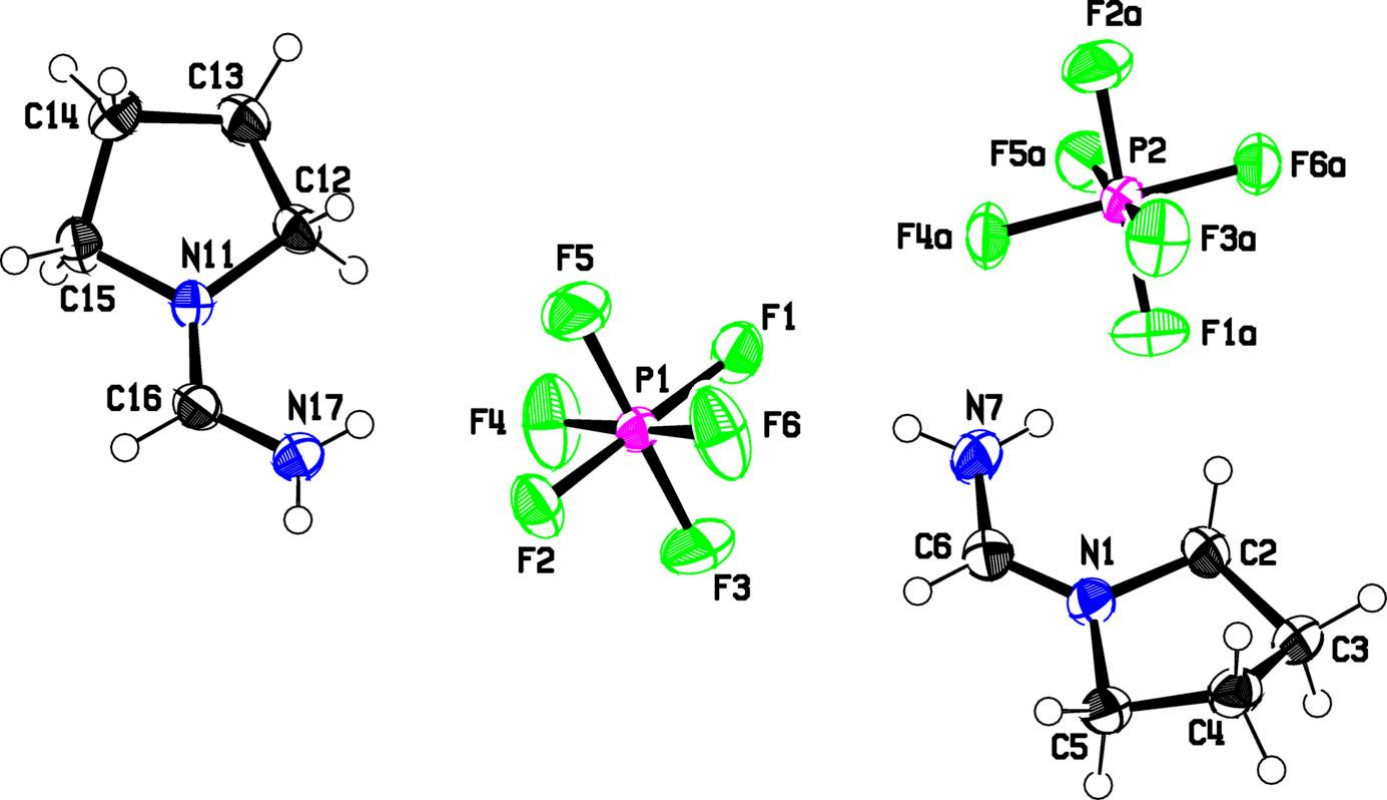
The asymmetric unit of **2**, showing the atom numbering. Displacement ellipsoids are drawn at the 50% probability level, displacement parameters for the H atoms are arbitrary, and atom colors as in Fig. 1 ▸.

Fig. 3 ▸ shows the mol­ecular structures of cations and anions for **2**. Here, the lengths of the delocalized C=N bonds in the two independent cations are slightly longer to the terminal nitro­gen atom: The average for the terminal C=N bonds, C6—N7 and C16—N17, is 1.323 (5) Å, while that for the C=N bonds adjacent to the rings, N1—C6 and N11—C16, is 1.293 (5) Å. A slight difference in the delocalized C=N bond lengths might be expected due to the differing inductive effects of the terminal H atoms and the ring atoms; the lower electron density expected on N1 in compound **1** due to the electron withdrawing inductive effect of the ring oxygen might cause the C=N distance adjacent to the ring to be longer in **1** than in **2**, and the terminal C=N distance to be shorter. The angles at the methine C6 and C16 atoms in **2** are 122.4 (5) and 123.1 (5)°, slightly smaller than in **1**. The five-membered pyrrolidine rings assume an envelope conformation with C4 and C14, respectively, as the flap (puckering parameters *Q*2 = 0.3982 Å, φ2 = 103.3° for the N1–C5 ring and *Q*2 = 0.3966, φ2 = 284.0° for the N11–C15 ring; Cremer & Pople, 1975 ▸). The envelope atoms N1, C2, C3, C5 and N11, C12, C13, C15 are coplanar, with deviations of 0.013 Å or less for both cations, and the C3, C4, C5 and C13, C14, C15 flaps make angles of 40.1 (4) and 39.6 (5)°, respectively, with these planes.

## 3. Supra­molecular features

Multiple contacts between the cations and the PF_6_^−^ anions may be due to either electrostatic or hydrogen-bonding inter­actions. We have applied a 3.25 Å cutoff for C/N ⋯ F distances and a 110^o^ C/N—H ⋯ F angle for possible hydrogen bonds and these inter­actions are listed in Tables 1 ▸–3 ▸ ▸.

**Figure 4:**
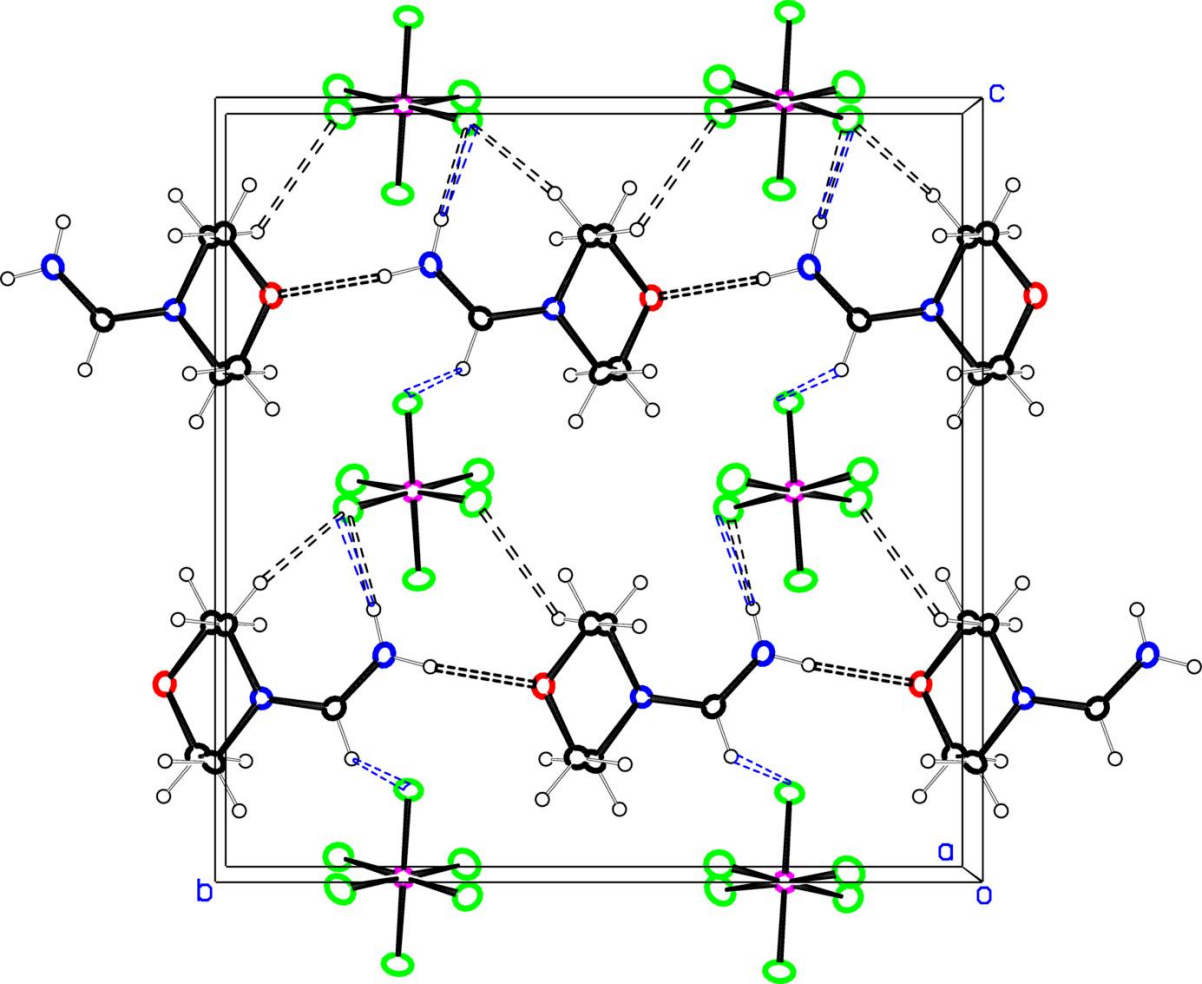
Projection of about half of the unit cell of **1** down the *a* axis. The minor disorder components for the PF_6_^−^ groups are not shown. The black dotted lines indicate hydrogen bonds in the layer shown, while the blue dotted lines indicate hydrogen bonds to anions half a cell above or below the layer shown. Atom colors are as in Fig. 1 ▸.

**Figure 5:**
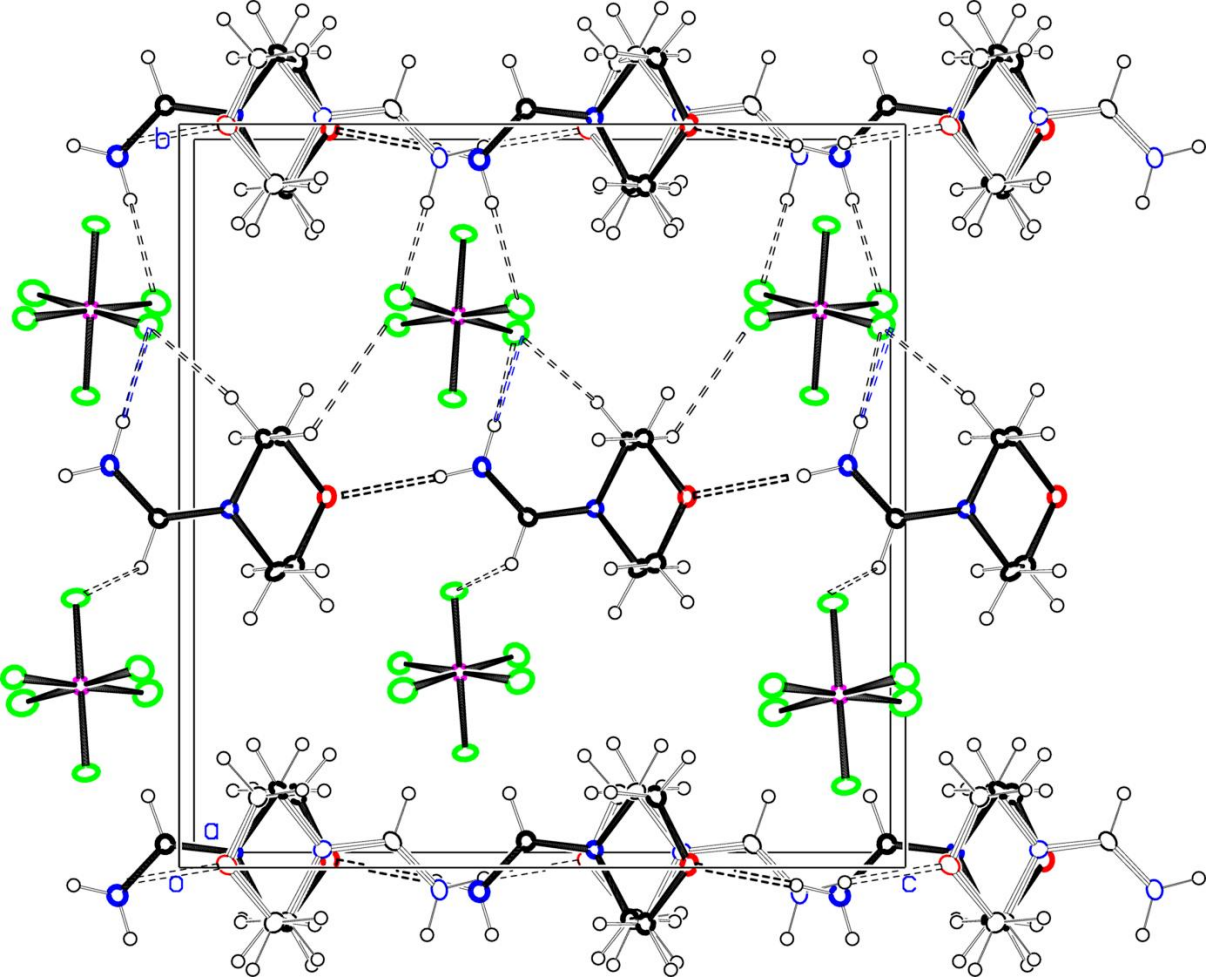
Projection of about half of the unit cell of **1**(recryst) down the *a* axis. The minor components for PF_6_^−^ groups are not shown. Colors of atoms and of hydrogen bonds are as in Fig. 1 ▸ and Fig. 4 ▸, respectively.

In **1**, the PF_6_^−^ anions are spaced close to half a unit cell apart in all three directions. There are chains of cations along the *b*-axis direction as seen in Fig. 4 ▸, linked together *via* N8—H8⋯O4 hydrogen bonds. N—H⋯F and C—H⋯F hydrogen bonds to PF_6_^−^ groups on one side of the cation chain augment these cation chains to ribbons of cations and anions parallel to the *b* axis. Cation chains at *z* = 1/4 and *z* = 3/4 are related by a *c* glide, and point in opposite directions. **1**(recryst) shows the same general supra­molecular features, Fig. 5 ▸, but in this case alternate cation chains are disordered as to direction, and the symmetry of the structure is lowered from the centric space group *Pbca* to the noncentric space group *Pca2_1_*, with an inter­change of the *b* and *c* axes. 36.1 (4)% of the chains point in a direction opposite to that of their neighbors, as would be required by the centric space group, but the majority disorder component points in the opposite direction. The network of cation⋯anion hydrogen bonds is similar to that in **1**, except that there do not appear to be any methine C—H⋯F contacts for either of the disordered cation chains. All inter­molecular H⋯H contacts in **1** are >2.7 Å.

**Figure 6:**
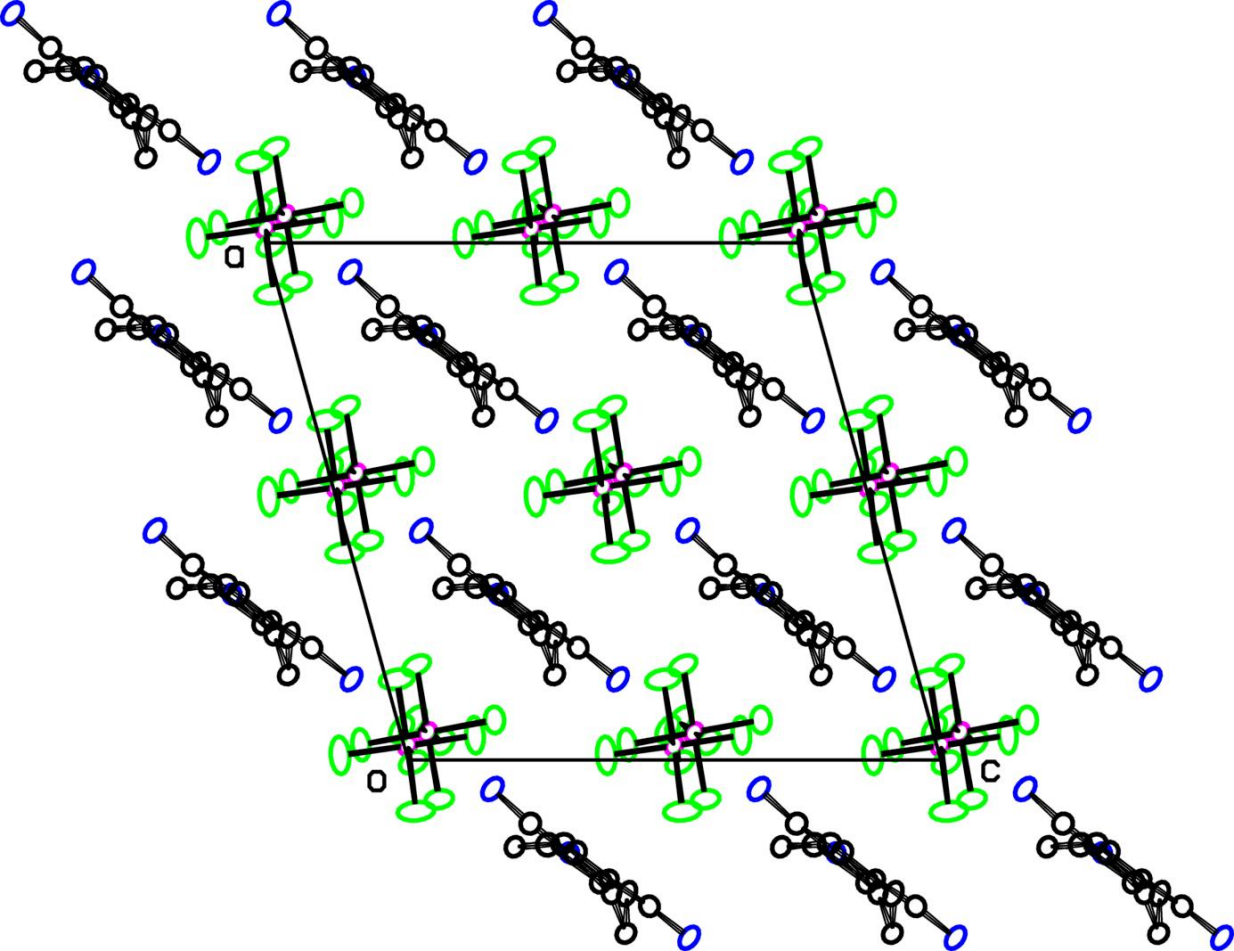
Projection of **2** down the *b* axis, showing the cations and anion components arranged in separate stacks along *b*. Atom colors are as in Fig. 1 ▸.

**Figure 7:**
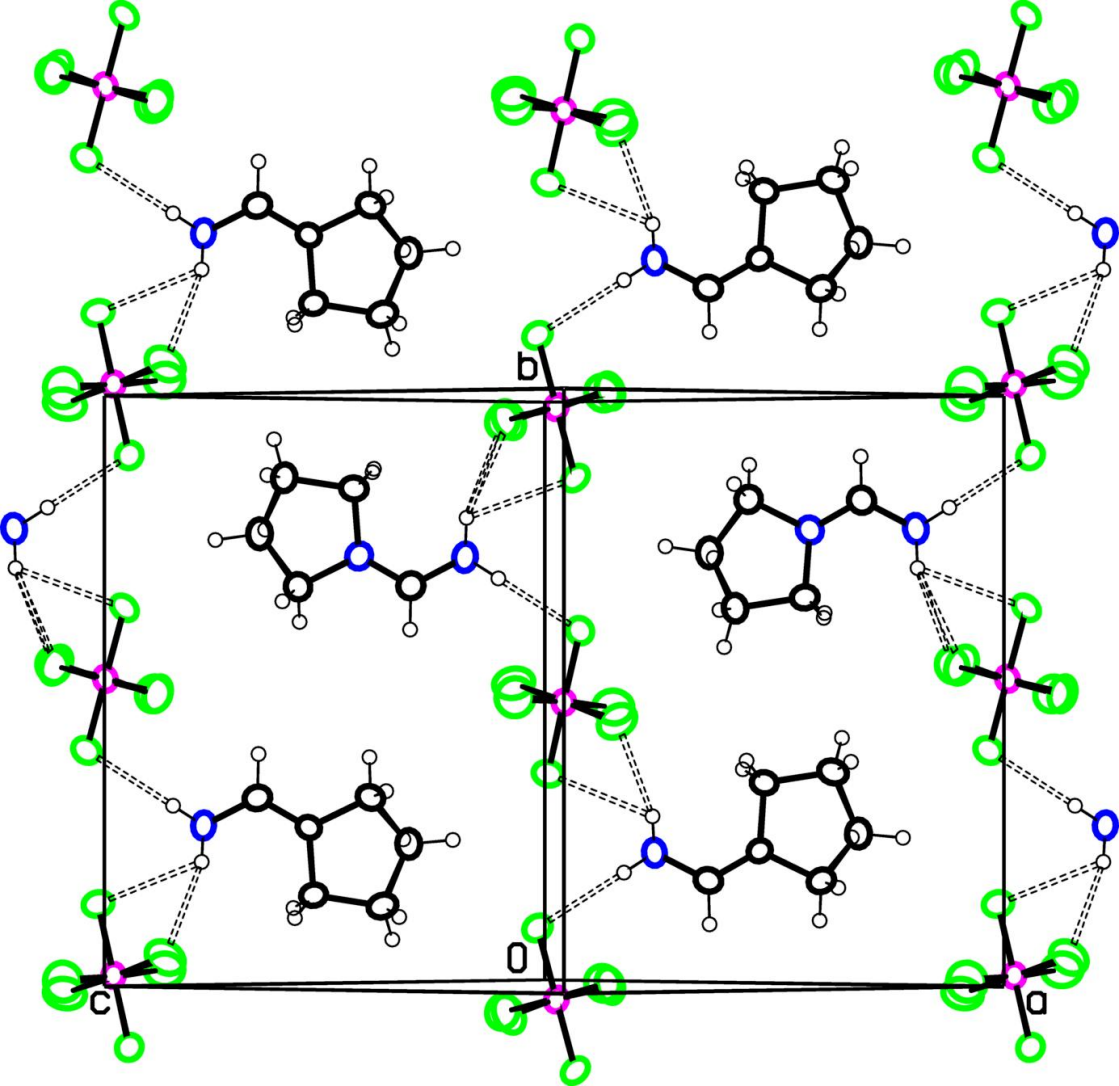
View of part of the structure of **2** projected approximately on (101), showing hydrogen-bonded cation⋯PF_6_⋯cation chains in the *b*-axis direction. Atom colors are as in Fig. 1 ▸.

In **2**, cations and anions are each spaced half a unit cell apart in all three directions as seen in Fig. 6 ▸. All but one of the N—H⋯F hydrogen-bonds listed in Table 3 ▸ are within sheets parallel to (101), and are shown for one of these sheets in Fig. 7 ▸ where hydrogen-bonded cation⋯anion⋯cation chains along the *b* axis can be seen. Alternate cations in the *b*-axis direction link to separate ribbons. The hydrogen-bonding pattern in Fig. 7 ▸ is not dissimilar to that for **1** shown in Fig. 4 ▸, except that the cation⋯cation hydrogen bonding in Fig. 4 ▸ is not possible in **2** due to lack of the O acceptor atom in the pyrrolidino ring. The C16—H16⋯F6 hydrogen bonds link the sheets together. The shortest H⋯H contacts are H2*A*⋯H13*A*(*x* − 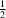 , 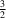 − *y*, *z* + 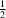 ) = 2.63 Å and H3*A*⋯H14*A*(*x* − 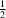 , *y* + 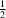 , *z* + 1) = 2.63 Å.

## 4. Database survey

Searches in the Cambridge Structural Database (CSD, version 5.43, update of October 2022; Groom *et al.*, 2016 ▸) with the fragment C—N—CH=NH_2_^+^ led to 17 hits, with just four different chemical species, *R*C(H/*R*_1_)NH_2_^+^, with *R* = Me_2_N—N=N–, CHO, and two more complex aromatic sulfur containing moieties. Overall, the delocalized C—N distances average to 1.310 (11) Å. Entry FUMGUP (Allenstein *et al.*, 1987 ▸) is the aldehyde derivative, where the delocalized C=N distances differ slightly, by 0.02 Å. We did not find any examples of terminal formamidinium groups attached to nitro­gen heterocycles, as in the present structures.

In regards to inter­molecular contacts, we found in the database 1825 N⋯F contacts less than 3.02 Å, the sum of the van der Waals radii. In the present compounds, only two N⋯F contact distances are less than 3.02 Å, while the others are all greater than this. Also, although there are over 35000 C⋯F contacts in the database less than 3.17 Å, the sum of the van der Waals radii, only the C7—H7⋯F2 contacts at 3.14 Å shown in Fig. 3 ▸ meet this criterion. The weak inter­molecular forces implied by the longer inter­molecular distances in the present crystal structures may be correlated with the disorder in the anions, and the disorder possibilities in the cation chains.

## 5. Synthesis and crystallization

Compound **1** was prepared by heating an equimolar mixture of morpholine, triethyl orthoformate and ammonium hexa­fluorido­phosphate. Similarly, compound **2** was made by heating an equimolar mixture of pyrrolidine, triethyl orthoformate and ammonium hexa­fluorido­phosphate. Compound **1** precipitated out as the reaction mixture was being heated and was purified by crystallization from ethanol. Compound **2** crystallized as the reaction mixture was cooled, affording sufficiently pure crystals.

**Infrared Spectra**: FTIR spectra for the two compounds are shown in the supporting information. For compound **2**, there are two clear NH_2_ stretching frequencies at 3474 and 3380 cm^−1^. The bands at 1716 cm^−1^ may be due to the resonant N—C=N stretches. For compound **1** and **1**(recyst), a similar N—C=N stretching frequency is seen at 1717 cm^−1^. Here, however, the spectrum in the N—H stretch region is more complex, with multiple bands below the prominent band at 3453 cm^−1^. Allenstein *et al.* (1987 ▸) include a review of the IR data for their aldehyde complex, with N—H stretches at 3342 and 3240 cm^−1^, and a band at 1695 cm^−1^ for the asymmetric N—C=N stretch; further assignments are given in more detail than covered in the present paper.

**Nuclear Magnetic Resonance Data: Compound 1**:

^1^H NMR δ (400 MHz, DMSO-*d*6): 3.50–3.73 (*m*, 8H), 8.10 (*s*, 1H), 8.85 (*s*, *br*, 2H).

**Compound 2**: ^1^H NMR δ (400 MHz, DMSO-*d*_6_): 1.80–2.05 (*m*, 4H), 3.25–3.40 (*t*, 2H), 3.60–3.69 (*t*, 2H), 8.10 (*s*, 1H), 8.70 (*s*, *br*, 2H).

## 6. Refinement

Crystal data, data collection and structure refinement details are summarized in Table 4 ▸. Methyl­ene H atoms were constrained to expected positions with C—H distances of 0.97 Å and displacement parameters set at 1.5*U*_eq_ of the parent C atom for **1** and **2**, and 1.2*U*_eq_ for **1**(recryst). H atoms bonded to N and the methine C atom were refined for **1**. For **1**(recryst) they were constrained due to the disorder [occupancy ratio of the disordered cation 0.639 (4):0.361 (4)], and they were also constrained for **2**, since refinements did not move them from their expected positions. N—H distances in **1**(recryst) and **2** were refined, however. Structure **1**(recryst) was refined as an inversion twin, although it seems more likely that the crystal had twinning about a mirror plane perpendicular to the *c* axis. Either twin operation has the same effect on the data analysis.

Initially, several sets of data were collected at room temperature on crystals of both compounds **1** and **2**. Room temperature data from all crystals had very few intensities with *I* > 2*σ*(*I*) at higher angles. The positions of the PF_6_^−^ groups, which dominate the X-ray scattering, lead to whole groups of weak reflections. Even though the room-temperature data were not sufficiently adequate to define the disorder in **1**(recryst), there were clear indications that the structure was not the same as in **1**: *R*_int_ for merged data from **1** and **1**(recryst) was 16.4%, compared with *R*_int_ values of 3.9% and 4.1% for the individual data sets. For this reason, data collection was repeated at low temperature.

Refinement was complicated by disorder in the hexa­fluorido­phosphate groups. In **1**, a minor disorder component was twisted some 45^o^ about the F1*A*—P1—F2*A* axis; since the occupancy of this component refined to only 13.0 (8)%, the four F atoms F3*B*–F6*B* were refined isotropically. A similar positional disorder exists for one of the PF_6_^−^ groups in **1**(recryst) with an occupancy ratio of 0.876 (19):0.124 (19), where the four F atoms F3*B*–F6*B* were refined isotropically. In **2**, no disordered model appeared necessary.

## Supplementary Material

Crystal structure: contains datablock(s) 1, 1recryst, 2. DOI: 10.1107/S2056989023010848/wm5706sup1.cif

Structure factors: contains datablock(s) 1. DOI: 10.1107/S2056989023010848/wm57061sup2.hkl

Structure factors: contains datablock(s) 1recryst. DOI: 10.1107/S2056989023010848/wm57061recrystsup3.hkl

Structure factors: contains datablock(s) 2. DOI: 10.1107/S2056989023010848/wm57062sup4.hkl

Infrared Spectra of the Two Compounds. DOI: 10.1107/S2056989023010848/wm5706sup5.tif

CCDC references: 2320366, 2320365, 2320364

Additional supporting information:  crystallographic information; 3D view; checkCIF report

## Figures and Tables

**Figure 2:**
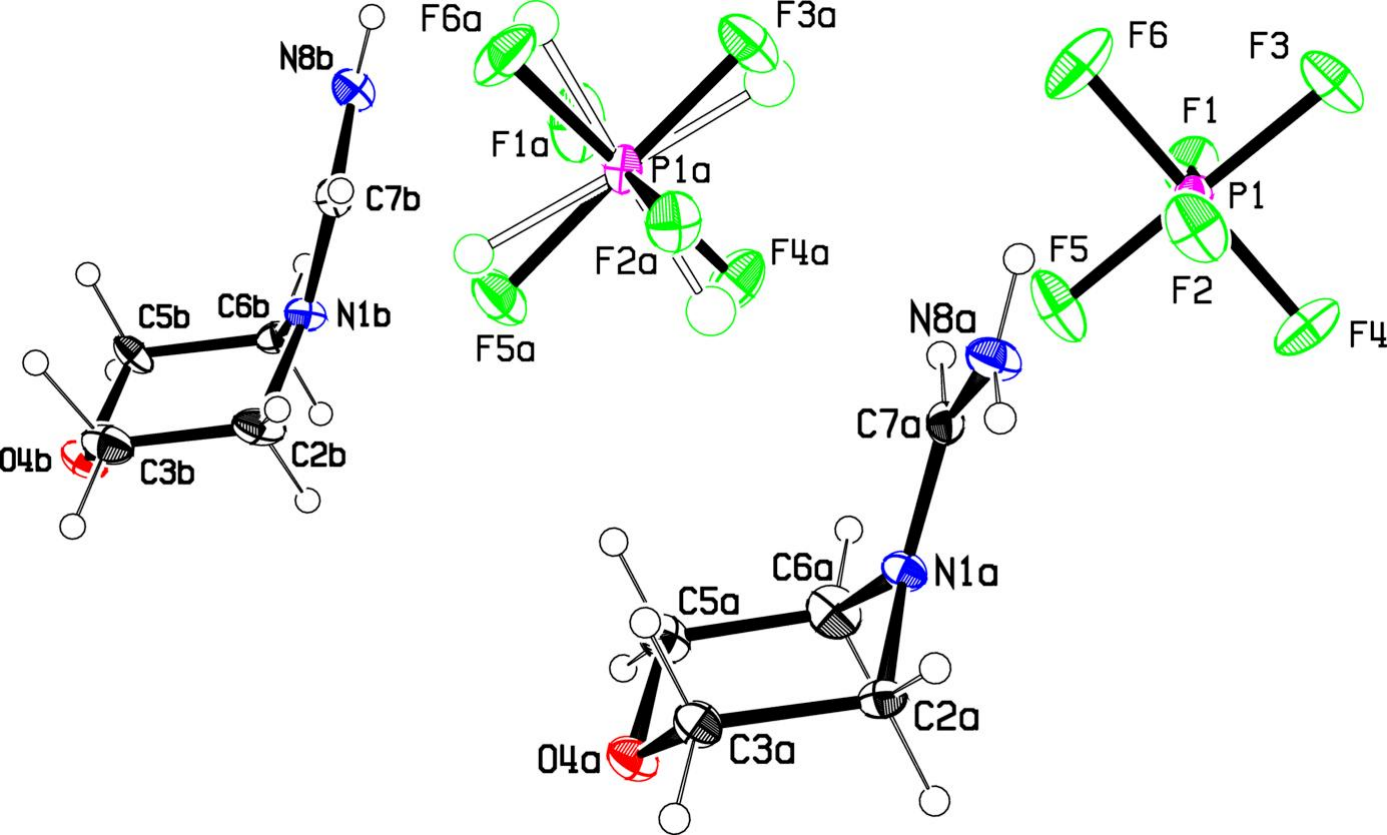
The asymmetric unit of **1**(recryst), showing the atom numbering. Displacement ellipsoids are drawn at the 50% probability level, while displacement parameters for the H atoms are arbitrary. The minor disordered PF_6_^−^ component is shown fa­inter. Colors are as in Fig. 1 ▸.

**Table 1:** Hydrogen-bond geometry (Å, °) for **1** [Chem scheme1]

*D*—H⋯*A*	*D*—H	H⋯*A*	*D*⋯*A*	*D*—H⋯*A*
C2—H2*A*⋯F4*A* ^i^	0.99	2.62	3.183 (2)	116
C2—H2*A*⋯F4*B* ^i^	0.99	2.53	3.126 (11)	119
C2—H2*B*⋯F3*A* ^ii^	0.99	2.37	3.1290 (19)	133
C7—H7⋯F2*A* ^iii^	0.946 (14)	2.522 (13)	3.0711 (12)	117.1 (9)
N8—H8*A*⋯F3*A* ^ii^	0.827 (18)	2.428 (17)	3.141 (2)	144.9 (13)
N8—H8*A*⋯F5*A* ^iv^	0.827 (18)	2.425 (16)	3.0699 (16)	135.4 (13)
N8—H8*A*⋯F3*B* ^ii^	0.827 (18)	2.36 (2)	3.105 (12)	150.1 (14)
N8—H8*A*⋯F5*B* ^iv^	0.827 (18)	2.331 (18)	3.031 (6)	142.8 (13)
N8—H8*B*⋯O4^v^	0.850 (18)	2.018 (18)	2.8572 (13)	168.8 (16)

Symmetry codes: (i) 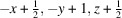 ; (ii) 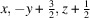 ; (iii) 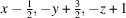 ; (iv) 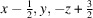 ; (v) 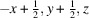 .

**Table 2:** Hydrogen-bond geometry (Å, °) for **1**(recryst)

*D*—H⋯*A*	*D*—H	H⋯*A*	*D*⋯*A*	*D*—H⋯*A*
C2*A*—H2*A*1⋯F4^i^	0.99	2.36	3.115 (3)	133
C2*A*—H2*A*2⋯F3^ii^	0.99	2.63	3.189 (3)	116
C6*A*—H6*A*2⋯F4*B*	0.99	2.42	3.17 (2)	132
C7*A*—H7*A*⋯F2	0.95	2.53	3.043 (3)	114
N8*A*—H8*A*1⋯F4^i^	0.83	2.40	3.096 (3)	142
N8*A*—H8*A*1⋯F5*A* ^iii^	0.83	2.45	3.135 (4)	140
N8*A*—H8*A*1⋯F5*B* ^iii^	0.83	2.33	3.043 (15)	144
N8*A*—H8*A*2⋯O4*A* ^iv^	0.83	2.04	2.864 (3)	169
N8*B*—H8*B*1⋯F5^v^	0.84	2.45	3.153 (4)	141
N8*B*—H8*B*2⋯O4*B* ^vi^	0.84	2.02	2.855 (5)	169
N8*B*′—H8*C*3⋯F6^vii^	0.88	2.34	3.028 (8)	135
N8*B*′—H8*C*4⋯O4*B*′^viii^	0.88	1.97	2.839 (12)	168

Symmetry codes: (i) 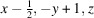 ; (ii) 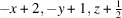 ; (iii) 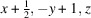 ; (iv) 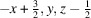 ; (v) 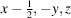 ; (vi) 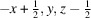 ; (vii) 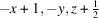 ; (viii) 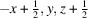 .

**Table 3:** Hydrogen-bond geometry (Å, °) for **2** [Chem scheme1]

*D*—H⋯*A*	*D*—H	H⋯*A*	*D*⋯*A*	*D*—H⋯*A*
N7—H7*B*⋯F1	0.86	2.11	2.966 (5)	177
N7—H7*A*⋯F3*A*	0.86	2.47	3.207 (6)	145
N7—H7*A*⋯F4*A*	0.86	2.51	2.945 (6)	112
C16—H16⋯F6^i^	0.95	2.54	3.153 (6)	122
N17—H17*A*⋯F2	0.79	2.51	2.935 (5)	115
N17—H17*A*⋯F4	0.79	2.30	3.026 (6)	152
N17—H17*B*⋯F6*A* ^ii^	0.79	2.23	3.019 (5)	179

Symmetry codes: (i) 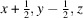 ; (ii) 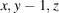 .

**Table 4:** Experimental details

	**1**	**1**(recryst)	**2**
Crystal data			
Chemical formula	C_5_H_11_N_2_O^+^·F_6_P^−^	C_5_H_11_N_2_O^+^·F_6_P^−^	C_5_H_11_N_2_^+^·F_6_P^−^
*M*_r_	260.13	260.13	244.13
Crystal system, space group	Orthorhombic, *P* *b* *c* *a*	Orthorhombic, *P* *c* *a*2_1_	Monoclinic, *C* *c*
Temperature (K)	130	100	130
*a*, *b*, *c* (Å)	10.4638 (3), 13.4495 (4), 13.7340 (4)	10.4504 (14), 13.7170 (16), 13.4157 (14)	12.3588 (3), 12.7942 (3), 12.2759 (3)
α, β, γ (°)	90, 90, 90	90, 90, 90	90, 105.400 (1), 90
*V* (Å^3^)	1932.83 (10)	1923.1 (4)	1871.38 (8)
*Z*	8	8	8
Radiation type	Cu *K*α	Mo *K*α	Cu *K*α
μ (mm^−1^)	3.30	0.36	3.28
Crystal size (mm)	0.29 × 0.09 × 0.06	0.24 × 0.23 × 0.14	0.21 × 0.21 × 0.11

Data collection			
Diffractometer	Bruker D8 with PHOTON III area detector	Bruker D8 Venture dual source	Bruker D8 with PHOTON III area detector
Absorption correction	Multi-scan (*SADABS*; Krause *et al.*, 2015 ▸)	Multi-scan (*SADABS*; Krause *et al.*, 2015 ▸)	Multi-scan (*SADABS*; Krause *et al.*, 2015 ▸)
*T*_min_, *T*_max_	0.570, 0.754	0.856, 0.971	0.639, 0.754
No. of measured, independent and observed [*I* > 2σ(*I*)] reflections	36889, 1969, 1923	42268, 7300, 5163	17935, 3467, 3450
*R*_int_	0.034	0.040	0.035
(sin θ/λ)_max_ (Å^−1^)	0.625	0.769	0.625

Refinement			
*R*[*F* ^2^ > 2σ(*F* ^2^)], *wR*(*F* ^2^), *S*	0.025, 0.071, 1.12	0.032, 0.088, 1.02	0.049, 0.135, 1.07
No. of reflections	1969	7300	3467
No. of parameters	165	364	257
No. of restraints	0	154	2
H-atom treatment	H atoms treated by a mixture of independent and constrained refinement	H atoms treated by a mixture of independent and constrained refinement	H atoms treated by a mixture of independent and constrained refinement
Δρ_max_, Δρ_min_ (e Å^−3^)	0.29, −0.28	0.28, −0.40	0.37, −0.35
Absolute structure	–	Twinning involves inversion, so Flack parameter cannot be determined	Refined as an inversion twin
Absolute structure parameter	–	0.5	0.49 (4)

Computer programs: *APEX4* (Bruker, 2016 ▸, 2022 ▸), *SAINT* (Bruker, 2022 ▸), *SHELXT* (Sheldrick, 2015*a* ▸), *SHELXL* (Sheldrick, 2015*b* ▸), *XP* in *SHELXTL* (Sheldrick, 2008 ▸), *WinGX* (Farrugia, 2012 ▸), *SHELX* (Sheldrick, 2008 ▸) and *publCIF* (Westrip, 2010 ▸).

## supplementary crystallographic information

### Morpholinoformamidinium hexafluorophosphate (1) Crystal data

**Table:**

C_5_H_11_N_2_O^+^·PF_6_^−^	*D*_x_ = 1.788 Mg m^−^^3^
*M**_r_* = 260.13	Cu *K*α radiation, λ = 1.54178 Å
Orthorhombic, *P**b**c**a*	Cell parameters from 9781 reflections
*a* = 10.4638 (3) Å	θ = 4.2–74.6°
*b* = 13.4495 (4) Å	µ = 3.30 mm^−^^1^
*c* = 13.7340 (4) Å	*T* = 130 K
*V* = 1932.83 (10) Å^3^	Block, colorless
*Z* = 8	0.29 × 0.09 × 0.06 mm
*F*(000) = 1056	

### Morpholinoformamidinium hexafluorophosphate (1) Data collection

**Table:**

Bruker D8 with PHOTON III area detector diffractometer	1923 reflections with *I* > 2σ(*I*)
Radiation source: microfocus	*R*_int_ = 0.034
φ and ω shutterless scans	θ_max_ = 74.6°, θ_min_ = 6.3°
Absorption correction: multi-scan (SADABS; Krause *et al.*, 2015)	*h* = −13→12
*T*_min_ = 0.570, *T*_max_ = 0.754	*k* = −16→16
36889 measured reflections	*l* = −17→15
1969 independent reflections	

### Morpholinoformamidinium hexafluorophosphate (1) Refinement

**Table:**

Refinement on *F*^2^	Primary atom site location: dual
Least-squares matrix: full	H atoms treated by a mixture of independent and constrained refinement
*R*[*F*^2^ > 2σ(*F*^2^)] = 0.025	*w* = 1/[σ^2^(*F*_o_^2^) + (0.0373*P*)^2^ + 0.517*P*] where *P* = (*F*_o_^2^ + 2*F*_c_^2^)/3
*wR*(*F*^2^) = 0.071	(Δ/σ)_max_ < 0.001
*S* = 1.12	Δρ_max_ = 0.29 e Å^−^^3^
1969 reflections	Δρ_min_ = −0.28 e Å^−^^3^
165 parameters	Extinction correction: SHELXL-2018/3 (Sheldrick, 2015b), Fc^*^=kFc[1+0.001xFc^2^λ^3^/sin(2θ)]^-1/4^
0 restraints	Extinction coefficient: 0.00052 (10)

### Morpholinoformamidinium hexafluorophosphate (1) Special details

**Table:**

Geometry. All esds (except the esd in the dihedral angle between two l.s. planes) are estimated using the full covariance matrix. The cell esds are taken into account individually in the estimation of esds in distances, angles and torsion angles; correlations between esds in cell parameters are only used when they are defined by crystal symmetry. An approximate (isotropic) treatment of cell esds is used for estimating esds involving l.s. planes.

### Morpholinoformamidinium hexafluorophosphate (1) Fractional atomic coordinates and isotropic or equivalent isotropic displacement parameters (Å^2^)

**Table:**

	*x*	*y*	*z*	*U*_iso_*/*U*_eq_	Occ. (<1)
N1	0.17230 (9)	0.55859 (6)	0.73256 (6)	0.01938 (19)	
C2	0.20187 (10)	0.51175 (7)	0.82640 (7)	0.0203 (2)	
H2A	0.152903	0.449208	0.833665	0.030*	
H2B	0.178416	0.556828	0.880516	0.030*	
C3	0.34450 (10)	0.49023 (8)	0.82874 (8)	0.0220 (2)	
H3A	0.392565	0.553533	0.825341	0.033*	
H3B	0.366643	0.456964	0.890787	0.033*	
O4	0.38048 (9)	0.42787 (6)	0.74877 (5)	0.02532 (19)	
C5	0.34896 (11)	0.47236 (9)	0.65681 (8)	0.0279 (3)	
H5A	0.373554	0.426656	0.603505	0.042*	
H5B	0.398048	0.534754	0.648785	0.042*	
C6	0.20684 (12)	0.49485 (8)	0.64996 (8)	0.0266 (2)	
H6A	0.157068	0.432289	0.652136	0.040*	
H6B	0.187731	0.529177	0.587877	0.040*	
C7	0.14903 (9)	0.65374 (8)	0.71986 (8)	0.0198 (2)	
H7	0.1387 (12)	0.6771 (10)	0.6554 (10)	0.023 (3)*	
N8	0.13576 (10)	0.71972 (7)	0.78845 (7)	0.0252 (2)	
H8A	0.1442 (14)	0.7058 (12)	0.8468 (13)	0.035 (3)*	
H8B	0.1207 (16)	0.7800 (13)	0.7735 (12)	0.035 (3)*	
P1A	0.39982 (2)	0.74360 (2)	0.49827 (2)	0.01852 (12)	0.870 (8)
F1A	0.36762 (8)	0.75062 (5)	0.61181 (5)	0.03015 (19)	0.870 (8)
F2A	0.43172 (8)	0.73592 (6)	0.38461 (5)	0.0350 (2)	0.870 (8)
F3A	0.2971 (3)	0.82864 (17)	0.47542 (12)	0.0382 (4)	0.870 (8)
F4A	0.2907 (2)	0.66066 (17)	0.48512 (12)	0.0372 (4)	0.870 (8)
F5A	0.5015 (2)	0.65703 (19)	0.52252 (9)	0.0372 (5)	0.870 (8)
F6A	0.5081 (2)	0.82470 (19)	0.51297 (13)	0.0418 (5)	0.870 (8)
P1B	0.39982 (2)	0.74360 (2)	0.49827 (2)	0.01852 (12)	0.130 (8)
F1B	0.36762 (8)	0.75062 (5)	0.61181 (5)	0.03015 (19)	0.130 (8)
F2B	0.43172 (8)	0.73592 (6)	0.38461 (5)	0.0350 (2)	0.130 (8)
F3B	0.2743 (11)	0.8078 (9)	0.4866 (8)	0.028 (2)*	0.130 (8)
F4B	0.3249 (11)	0.6487 (8)	0.4898 (7)	0.024 (2)*	0.130 (8)
F5B	0.5314 (7)	0.6899 (8)	0.5077 (6)	0.0209 (18)*	0.130 (8)
F6B	0.4809 (8)	0.8509 (7)	0.4946 (6)	0.0246 (19)*	0.130 (8)

### Morpholinoformamidinium hexafluorophosphate (1) Atomic displacement parameters (Å^2^)

**Table:**

	*U*^11^	*U*^22^	*U*^33^	*U*^12^	*U*^13^	*U*^23^
N1	0.0225 (4)	0.0178 (4)	0.0179 (4)	−0.0003 (3)	−0.0010 (3)	−0.0006 (3)
C2	0.0246 (5)	0.0177 (4)	0.0187 (4)	−0.0004 (4)	0.0013 (4)	0.0030 (4)
C3	0.0247 (5)	0.0201 (5)	0.0213 (5)	0.0014 (4)	−0.0003 (4)	0.0000 (4)
O4	0.0317 (4)	0.0189 (4)	0.0254 (4)	0.0059 (3)	0.0032 (3)	0.0006 (3)
C5	0.0371 (6)	0.0250 (5)	0.0215 (5)	0.0069 (4)	0.0064 (4)	0.0000 (4)
C6	0.0367 (6)	0.0230 (5)	0.0200 (5)	0.0016 (4)	−0.0026 (4)	−0.0051 (4)
C7	0.0169 (5)	0.0214 (5)	0.0212 (5)	0.0003 (4)	−0.0001 (4)	0.0030 (4)
N8	0.0330 (5)	0.0180 (5)	0.0246 (5)	0.0048 (4)	0.0020 (4)	0.0018 (4)
P1A	0.01731 (18)	0.02057 (17)	0.01767 (17)	−0.00065 (9)	0.00018 (9)	−0.00186 (8)
F1A	0.0365 (4)	0.0350 (4)	0.0190 (3)	0.0065 (3)	0.0023 (3)	−0.0025 (2)
F2A	0.0409 (4)	0.0441 (4)	0.0202 (3)	−0.0083 (3)	0.0070 (3)	−0.0029 (3)
F3A	0.0463 (9)	0.0355 (8)	0.0329 (6)	0.0184 (7)	−0.0065 (6)	0.0044 (6)
F4A	0.0325 (8)	0.0400 (7)	0.0392 (6)	−0.0187 (7)	0.0066 (6)	−0.0102 (5)
F5A	0.0391 (7)	0.0389 (9)	0.0336 (5)	0.0200 (7)	0.0017 (5)	−0.0011 (5)
F6A	0.0385 (7)	0.0446 (9)	0.0423 (6)	−0.0229 (7)	0.0000 (6)	−0.0070 (6)
P1B	0.01731 (18)	0.02057 (17)	0.01767 (17)	−0.00065 (9)	0.00018 (9)	−0.00186 (8)
F1B	0.0365 (4)	0.0350 (4)	0.0190 (3)	0.0065 (3)	0.0023 (3)	−0.0025 (2)
F2B	0.0409 (4)	0.0441 (4)	0.0202 (3)	−0.0083 (3)	0.0070 (3)	−0.0029 (3)

### Morpholinoformamidinium hexafluorophosphate (1) Geometric parameters (Å, º)

**Table:**

N1—C7	1.3142 (13)	C7—H7	0.946 (14)
N1—C2	1.4675 (12)	N8—H8A	0.827 (18)
N1—C6	1.4672 (13)	N8—H8B	0.850 (18)
C2—C3	1.5206 (14)	P1A—F6A	1.5856 (10)
C2—H2A	0.9900	P1A—F3A	1.6003 (14)
C2—H2B	0.9900	P1A—F4A	1.6063 (14)
C3—O4	1.4322 (12)	P1A—F5A	1.6120 (11)
C3—H3A	0.9900	P1A—F1A	1.5982 (7)
C3—H3B	0.9900	P1A—F2A	1.5997 (7)
O4—C5	1.4360 (13)	P1B—F4B	1.502 (12)
C5—C6	1.5204 (16)	P1B—F3B	1.581 (13)
C5—H5A	0.9900	P1B—F5B	1.560 (6)
C5—H5B	0.9900	P1B—F1B	1.5982 (7)
C6—H6A	0.9900	P1B—F2B	1.5997 (7)
C6—H6B	0.9900	P1B—F6B	1.674 (8)
C7—N8	1.3016 (15)		
			
C7—N1—C2	125.00 (9)	C7—N8—H8B	119.6 (11)
C7—N1—C6	120.79 (9)	H8A—N8—H8B	118.0 (15)
C2—N1—C6	112.10 (8)	F6A—P1A—F3A	90.75 (9)
N1—C2—C3	107.89 (8)	F6A—P1A—F4A	179.05 (8)
N1—C2—H2A	110.1	F3A—P1A—F4A	89.83 (10)
C3—C2—H2A	110.1	F6A—P1A—F5A	89.94 (9)
N1—C2—H2B	110.1	F3A—P1A—F5A	179.02 (8)
C3—C2—H2B	110.1	F4A—P1A—F5A	89.47 (8)
H2A—C2—H2B	108.4	F6A—P1A—F1A	89.19 (6)
O4—C3—C2	110.69 (8)	F3A—P1A—F1A	90.44 (7)
O4—C3—H3A	109.5	F4A—P1A—F1A	90.05 (6)
C2—C3—H3A	109.5	F5A—P1A—F1A	88.87 (6)
O4—C3—H3B	109.5	F6A—P1A—F2A	91.12 (6)
C2—C3—H3B	109.5	F3A—P1A—F2A	89.70 (7)
H3A—C3—H3B	108.1	F4A—P1A—F2A	89.64 (6)
C5—O4—C3	111.72 (8)	F5A—P1A—F2A	90.99 (6)
O4—C5—C6	111.22 (9)	F1A—P1A—F2A	179.66 (4)
O4—C5—H5A	109.4	F4B—P1B—F3B	91.3 (5)
C6—C5—H5A	109.4	F4B—P1B—F5B	94.2 (4)
O4—C5—H5B	109.4	F3B—P1B—F5B	174.3 (4)
C6—C5—H5B	109.4	F4B—P1B—F1B	90.9 (4)
H5A—C5—H5B	108.0	F3B—P1B—F1B	83.8 (4)
C5—C6—N1	108.02 (9)	F5B—P1B—F1B	97.6 (3)
C5—C6—H6A	110.1	F4B—P1B—F2B	88.8 (4)
N1—C6—H6A	110.1	F3B—P1B—F2B	96.3 (4)
C5—C6—H6B	110.1	F5B—P1B—F2B	82.4 (3)
N1—C6—H6B	110.1	F1B—P1B—F2B	179.66 (4)
H6A—C6—H6B	108.4	F4B—P1B—F6B	173.7 (5)
N1—C7—N8	125.98 (10)	F3B—P1B—F6B	87.0 (4)
N1—C7—H7	118.0 (8)	F5B—P1B—F6B	87.4 (4)
N8—C7—H7	116.0 (8)	F1B—P1B—F6B	94.9 (3)
C7—N8—H8A	122.4 (11)	F2B—P1B—F6B	85.4 (3)

### Morpholinoformamidinium hexafluorophosphate (1) Hydrogen-bond geometry (Å, º)

**Table:**

*D*—H···*A*	*D*—H	H···*A*	*D*···*A*	*D*—H···*A*
C2—H2*A*···F4*A*^i^	0.99	2.62	3.183 (2)	116
C2—H2*A*···F4*B*^i^	0.99	2.53	3.126 (11)	119
C2—H2*B*···F3*A*^ii^	0.99	2.37	3.1290 (19)	133
C7—H7···F2*A*^iii^	0.946 (14)	2.522 (13)	3.0711 (12)	117.1 (9)
N8—H8*A*···F3*A*^ii^	0.827 (18)	2.428 (17)	3.141 (2)	144.9 (13)
N8—H8*A*···F5*A*^iv^	0.827 (18)	2.425 (16)	3.0699 (16)	135.4 (13)
N8—H8*A*···F3*B*^ii^	0.827 (18)	2.36 (2)	3.105 (12)	150.1 (14)
N8—H8*A*···F5*B*^iv^	0.827 (18)	2.331 (18)	3.031 (6)	142.8 (13)
N8—H8*B*···O4^v^	0.850 (18)	2.018 (18)	2.8572 (13)	168.8 (16)

Symmetry codes: (i) −*x*+1/2, −*y*+1, *z*+1/2; (ii) *x*, −*y*+3/2, *z*+1/2; (iii) *x*−1/2, −*y*+3/2, −*z*+1; (iv) *x*−1/2, *y*, −*z*+3/2; (v) −*x*+1/2, *y*+1/2, *z*.

### Morpholinoformamidinium hexafluorophosphate (1recryst) Crystal data

**Table:**

C_5_H_11_N_2_O^+^·PF_6_^−^	*D*_x_ = 1.797 Mg m^−^^3^
*M**_r_* = 260.13	Mo *K*α radiation, λ = 0.71073 Å
Orthorhombic, *P**c**a*2_1_	Cell parameters from 9865 reflections
*a* = 10.4504 (14) Å	θ = 3.4–33.2°
*b* = 13.7170 (16) Å	µ = 0.36 mm^−^^1^
*c* = 13.4157 (14) Å	*T* = 100 K
*V* = 1923.1 (4) Å^3^	Block, colourless
*Z* = 8	0.24 × 0.23 × 0.14 mm
*F*(000) = 1056	

### Morpholinoformamidinium hexafluorophosphate (1recryst) Data collection

**Table:**

Bruker D8 Venture dual source diffractometer	7300 independent reflections
Radiation source: microsource	5163 reflections with *I* > 2σ(*I*)
Detector resolution: 7.41 pixels mm^-1^	*R*_int_ = 0.040
φ and ω scans	θ_max_ = 33.1°, θ_min_ = 2.0°
Absorption correction: multi-scan (SADABS; Krause *et al.*, 2015)	*h* = −16→16
*T*_min_ = 0.856, *T*_max_ = 0.971	*k* = −21→20
42268 measured reflections	*l* = −20→20

### Morpholinoformamidinium hexafluorophosphate (1recryst) Refinement

**Table:**

Refinement on *F*^2^	Hydrogen site location: inferred from neighbouring sites
Least-squares matrix: full	H atoms treated by a mixture of independent and constrained refinement
*R*[*F*^2^ > 2σ(*F*^2^)] = 0.032	*w* = 1/[σ^2^(*F*_o_^2^) + (0.039*P*)^2^ + 0.260*P*] where *P* = (*F*_o_^2^ + 2*F*_c_^2^)/3
*wR*(*F*^2^) = 0.088	(Δ/σ)_max_ = 0.016
*S* = 1.02	Δρ_max_ = 0.28 e Å^−^^3^
7300 reflections	Δρ_min_ = −0.40 e Å^−^^3^
364 parameters	Absolute structure: Twinning involves inversion, so Flack parameter cannot be determined
154 restraints	Absolute structure parameter: 0.5
Primary atom site location: structure-invariant direct methods	

### Morpholinoformamidinium hexafluorophosphate (1recryst) Special details

**Table:**

Geometry. All esds (except the esd in the dihedral angle between two l.s. planes) are estimated using the full covariance matrix. The cell esds are taken into account individually in the estimation of esds in distances, angles and torsion angles; correlations between esds in cell parameters are only used when they are defined by crystal symmetry. An approximate (isotropic) treatment of cell esds is used for estimating esds involving l.s. planes.
Refinement. Refined as a 2-component inversion twin.

### Morpholinoformamidinium hexafluorophosphate (1recryst) Fractional atomic coordinates and isotropic or equivalent isotropic displacement parameters (Å^2^)

**Table:**

	*x*	*y*	*z*	*U*_iso_*/*U*_eq_	Occ. (<1)
N1A	0.8290 (2)	0.48239 (16)	0.57523 (16)	0.0129 (4)	
C2A	0.8000 (2)	0.57601 (18)	0.6226 (2)	0.0140 (5)	
H2A1	0.824879	0.630306	0.578020	0.017*	
H2A2	0.848505	0.582406	0.685693	0.017*	
C3A	0.6569 (2)	0.57971 (19)	0.6434 (2)	0.0151 (5)	
H3A1	0.635556	0.641635	0.677442	0.018*	
H3A2	0.609306	0.577543	0.579648	0.018*	
O4A	0.6192 (2)	0.49898 (12)	0.70486 (17)	0.0177 (4)	
C5A	0.6500 (2)	0.40691 (18)	0.6598 (2)	0.0193 (5)	
H5A1	0.601492	0.399677	0.596823	0.023*	
H5A2	0.624178	0.353401	0.705039	0.023*	
C6A	0.7926 (3)	0.39933 (17)	0.63826 (19)	0.0193 (5)	
H6A1	0.841621	0.400702	0.701372	0.023*	
H6A2	0.811463	0.337321	0.603486	0.023*	
C7A	0.85190 (19)	0.47015 (19)	0.47963 (18)	0.0129 (4)	
H7A	0.858586	0.405015	0.456128	0.016*	
N8A	0.8664 (2)	0.54034 (17)	0.41380 (18)	0.0172 (4)	
H8A1	0.8612 (2)	0.5984 (15)	0.4316 (5)	0.021*	
H8A2	0.8807 (4)	0.5267 (3)	0.3543 (14)	0.021*	
N1B	0.3318 (4)	0.0173 (3)	0.5727 (3)	0.0140 (6)	0.639 (4)
C2B	0.2939 (4)	0.1008 (2)	0.6349 (3)	0.0194 (7)	0.639 (4)
H2B1	0.311558	0.162540	0.599144	0.023*	0.639 (4)
H2B2	0.343451	0.100825	0.697733	0.023*	0.639 (4)
C3B	0.1518 (4)	0.0928 (3)	0.6575 (3)	0.0215 (7)	0.639 (4)
H3B1	0.126171	0.146548	0.702529	0.026*	0.639 (4)
H3B2	0.102490	0.099394	0.594845	0.026*	0.639 (4)
O4B	0.1222 (3)	0.0012 (2)	0.7034 (2)	0.0191 (6)	0.639 (4)
C5B	0.1604 (5)	−0.0796 (6)	0.6432 (6)	0.0179 (10)	0.639 (4)
H5B1	0.112518	−0.078428	0.579499	0.022*	0.639 (4)
H5B2	0.139565	−0.141230	0.678005	0.022*	0.639 (4)
C6B	0.3031 (4)	−0.0758 (5)	0.6219 (5)	0.0143 (9)	0.639 (4)
H6B1	0.351804	−0.081037	0.684996	0.017*	0.639 (4)
H6B2	0.327946	−0.130785	0.578259	0.017*	0.639 (4)
C7B	0.3530 (3)	0.0287 (2)	0.4776 (2)	0.0149 (6)	0.639 (4)
H7B	0.359065	0.093865	0.454013	0.018*	0.639 (4)
N8B	0.3668 (3)	−0.0406 (2)	0.4117 (3)	0.0192 (6)	0.639 (4)
H8B1	0.3622 (3)	−0.099 (3)	0.4294 (8)	0.023*	0.639 (4)
H8B2	0.3804 (7)	−0.0264 (6)	0.352 (3)	0.023*	0.639 (4)
N1B'	0.1687 (8)	0.0176 (6)	0.6935 (6)	0.0199 (15)	0.361 (4)
C2B'	0.1990 (10)	−0.0769 (9)	0.6440 (12)	0.021 (2)	0.361 (4)
H2C3	0.154138	−0.081905	0.579227	0.025*	0.361 (4)
H2C4	0.172359	−0.132281	0.686577	0.025*	0.361 (4)
C3B'	0.3421 (9)	−0.0779 (10)	0.6288 (11)	0.022 (2)	0.361 (4)
H3C3	0.385858	−0.070707	0.693820	0.027*	0.361 (4)
H3C4	0.368428	−0.140949	0.599215	0.027*	0.361 (4)
O4B'	0.3787 (8)	0.0008 (5)	0.5636 (6)	0.0240 (15)	0.361 (4)
C5B'	0.3445 (8)	0.0923 (6)	0.6060 (7)	0.0241 (17)	0.361 (4)
H5C3	0.367287	0.144961	0.558796	0.029*	0.361 (4)
H5C4	0.394545	0.102477	0.667809	0.029*	0.361 (4)
C6B'	0.2036 (8)	0.0993 (5)	0.6303 (6)	0.0214 (16)	0.361 (4)
H6C3	0.185643	0.161482	0.665102	0.026*	0.361 (4)
H6C4	0.152664	0.097779	0.568073	0.026*	0.361 (4)
C7B'	0.1453 (6)	0.0285 (6)	0.7905 (5)	0.0185 (14)	0.361 (4)
H7B'	0.133998	0.093460	0.813339	0.022*	0.361 (4)
N8B'	0.1362 (7)	−0.0380 (5)	0.8561 (6)	0.0237 (14)	0.361 (4)
H8C3	0.145814	−0.099558	0.839021	0.028*	0.361 (4)
H8C4	0.120303	−0.022593	0.918522	0.028*	0.361 (4)
P1	1.10174 (10)	0.25140 (7)	0.38095 (4)	0.0133 (2)	
F1	1.1345 (2)	0.13771 (15)	0.38914 (11)	0.0236 (5)	
F2	1.0694 (2)	0.36564 (14)	0.37324 (14)	0.0266 (4)	
F3	1.2054 (2)	0.26335 (12)	0.29446 (17)	0.0293 (5)	
F4	1.2093 (2)	0.27439 (14)	0.46292 (18)	0.0319 (5)	
F5	0.9982 (2)	0.24007 (13)	0.46808 (17)	0.0344 (5)	
F6	0.9944 (2)	0.22733 (15)	0.29994 (19)	0.0364 (5)	
P1A	0.60169 (10)	0.24864 (7)	0.38572 (4)	0.0148 (2)	0.876 (19)
F1A	0.5698 (2)	0.13505 (16)	0.38354 (13)	0.0355 (6)	0.876 (19)
F2A	0.6341 (2)	0.36245 (16)	0.38891 (11)	0.0230 (5)	0.876 (19)
F3A	0.7033 (5)	0.2329 (2)	0.2977 (4)	0.0302 (8)	0.876 (19)
F4A	0.7122 (4)	0.2274 (3)	0.4658 (4)	0.0308 (7)	0.876 (19)
F5A	0.5008 (5)	0.2645 (2)	0.4755 (4)	0.0295 (8)	0.876 (19)
F6A	0.4912 (5)	0.2708 (3)	0.3067 (4)	0.0333 (8)	0.876 (19)
P1B	0.60169 (10)	0.24864 (7)	0.38572 (4)	0.0148 (2)	0.124 (19)
F1B	0.5698 (2)	0.13505 (16)	0.38354 (13)	0.0355 (6)	0.124 (19)
F2B	0.6341 (2)	0.36245 (16)	0.38891 (11)	0.0230 (5)	0.124 (19)
F3B	0.723 (2)	0.2384 (16)	0.3217 (19)	0.022 (4)*	0.124 (19)
F4B	0.690 (2)	0.2428 (18)	0.4841 (18)	0.022 (4)*	0.124 (19)
F5B	0.4767 (16)	0.2571 (11)	0.4483 (16)	0.013 (3)*	0.124 (19)
F6B	0.517 (2)	0.2531 (16)	0.2880 (14)	0.018 (4)*	0.124 (19)

### Morpholinoformamidinium hexafluorophosphate (1recryst) Atomic displacement parameters (Å^2^)

**Table:**

	*U*^11^	*U*^22^	*U*^33^	*U*^12^	*U*^13^	*U*^23^
N1A	0.0147 (9)	0.0120 (9)	0.0119 (9)	0.0017 (8)	0.0010 (8)	0.0001 (8)
C2A	0.0165 (12)	0.0132 (10)	0.0123 (10)	0.0002 (9)	−0.0010 (10)	−0.0029 (8)
C3A	0.0165 (12)	0.0139 (11)	0.0150 (11)	0.0013 (8)	0.0013 (9)	0.0003 (9)
O4A	0.0202 (9)	0.0196 (10)	0.0134 (8)	−0.0006 (7)	0.0041 (9)	0.0005 (6)
C5A	0.0232 (14)	0.0156 (12)	0.0192 (13)	−0.0044 (9)	0.0054 (9)	0.0008 (10)
C6A	0.0241 (14)	0.0143 (11)	0.0194 (13)	0.0023 (10)	0.0014 (10)	0.0061 (8)
C7A	0.0096 (9)	0.0144 (11)	0.0148 (10)	0.0003 (7)	0.0004 (7)	−0.0015 (9)
N8A	0.0219 (10)	0.0173 (10)	0.0123 (10)	−0.0012 (8)	0.0035 (9)	−0.0004 (9)
N1B	0.0179 (17)	0.0127 (13)	0.0114 (12)	−0.0012 (12)	0.0011 (13)	−0.0010 (10)
C2B	0.029 (2)	0.0117 (13)	0.0179 (17)	−0.0032 (14)	0.0032 (14)	−0.0058 (11)
C3B	0.029 (2)	0.0192 (16)	0.0169 (16)	0.0056 (14)	0.0024 (14)	−0.0024 (12)
O4B	0.0243 (17)	0.0200 (14)	0.0129 (11)	0.0027 (12)	0.0056 (12)	0.0022 (9)
C5B	0.018 (2)	0.0200 (18)	0.0155 (18)	0.000 (2)	0.009 (2)	0.0006 (13)
C6B	0.018 (2)	0.0133 (15)	0.0117 (15)	0.0008 (19)	−0.0031 (19)	0.0007 (11)
C7B	0.0128 (13)	0.0166 (13)	0.0153 (13)	0.0000 (10)	0.0009 (10)	−0.0001 (10)
N8B	0.0199 (14)	0.0193 (14)	0.0185 (14)	0.0009 (11)	0.0033 (12)	−0.0012 (11)
N1B'	0.024 (4)	0.017 (3)	0.019 (3)	−0.003 (3)	0.003 (3)	0.001 (2)
C2B'	0.028 (5)	0.011 (3)	0.024 (4)	−0.003 (4)	0.009 (5)	−0.001 (3)
C3B'	0.021 (5)	0.024 (4)	0.023 (5)	−0.002 (4)	0.001 (4)	0.002 (3)
O4B'	0.028 (4)	0.021 (3)	0.024 (3)	−0.005 (3)	0.008 (3)	−0.004 (2)
C5B'	0.030 (4)	0.023 (4)	0.020 (4)	−0.002 (3)	0.006 (3)	0.002 (3)
C6B'	0.029 (4)	0.014 (3)	0.021 (4)	0.002 (3)	0.012 (3)	0.002 (3)
C7B'	0.017 (3)	0.020 (3)	0.019 (3)	−0.003 (2)	0.001 (2)	−0.005 (3)
N8B'	0.033 (4)	0.023 (3)	0.014 (3)	−0.001 (3)	0.005 (3)	−0.001 (3)
P1	0.0120 (5)	0.0128 (4)	0.0152 (4)	0.0003 (3)	0.00030 (18)	−0.00023 (16)
F1	0.0264 (12)	0.0121 (10)	0.0323 (13)	0.0020 (9)	−0.0029 (6)	0.0027 (5)
F2	0.0299 (11)	0.0138 (9)	0.0360 (8)	0.0056 (8)	0.0105 (7)	0.0027 (6)
F3	0.0357 (12)	0.0234 (8)	0.0287 (11)	0.0025 (7)	0.0191 (9)	0.0025 (8)
F4	0.0334 (11)	0.0295 (9)	0.0328 (11)	−0.0075 (8)	−0.0162 (9)	−0.0048 (8)
F5	0.0306 (11)	0.0319 (10)	0.0408 (14)	0.0003 (9)	0.0209 (11)	0.0066 (9)
F6	0.0336 (11)	0.0311 (9)	0.0445 (14)	−0.0029 (8)	−0.0246 (11)	0.0029 (9)
P1A	0.0115 (5)	0.0135 (4)	0.0193 (6)	−0.0004 (3)	−0.00038 (17)	0.00138 (15)
F1A	0.0291 (12)	0.0153 (10)	0.0620 (16)	−0.0064 (9)	0.0038 (8)	−0.0005 (7)
F2A	0.0240 (12)	0.0152 (11)	0.0297 (12)	−0.0021 (9)	0.0008 (5)	−0.0001 (5)
F3A	0.0329 (17)	0.0286 (12)	0.0290 (16)	−0.0004 (10)	0.0151 (14)	−0.0042 (11)
F4A	0.0258 (13)	0.0334 (13)	0.0333 (16)	0.0055 (10)	−0.0139 (11)	0.0052 (12)
F5A	0.0275 (14)	0.0349 (12)	0.0262 (16)	−0.0025 (10)	0.0128 (14)	−0.0002 (11)
F6A	0.0281 (14)	0.0353 (13)	0.0366 (15)	0.0069 (11)	−0.0193 (12)	−0.0072 (12)
P1B	0.0115 (5)	0.0135 (4)	0.0193 (6)	−0.0004 (3)	−0.00038 (17)	0.00138 (15)
F1B	0.0291 (12)	0.0153 (10)	0.0620 (16)	−0.0064 (9)	0.0038 (8)	−0.0005 (7)
F2B	0.0240 (12)	0.0152 (11)	0.0297 (12)	−0.0021 (9)	0.0008 (5)	−0.0001 (5)

### Morpholinoformamidinium hexafluorophosphate (1recryst) Geometric parameters (Å, º)

**Table:**

N1A—C7A	1.315 (3)	N1B'—C7B'	1.333 (10)
N1A—C2A	1.465 (3)	N1B'—C6B'	1.452 (10)
N1A—C6A	1.469 (3)	N1B'—C2B'	1.492 (14)
C2A—C3A	1.522 (3)	C2B'—C3B'	1.509 (12)
C2A—H2A1	0.9900	C2B'—H2C3	0.9900
C2A—H2A2	0.9900	C2B'—H2C4	0.9900
C3A—O4A	1.436 (3)	C3B'—O4B'	1.442 (13)
C3A—H3A1	0.9900	C3B'—H3C3	0.9900
C3A—H3A2	0.9900	C3B'—H3C4	0.9900
O4A—C5A	1.437 (3)	O4B'—C5B'	1.424 (10)
C5A—C6A	1.521 (3)	C5B'—C6B'	1.511 (10)
C5A—H5A1	0.9900	C5B'—H5C3	0.9900
C5A—H5A2	0.9900	C5B'—H5C4	0.9900
C6A—H6A1	0.9900	C6B'—H6C3	0.9900
C6A—H6A2	0.9900	C6B'—H6C4	0.9900
C7A—N8A	1.315 (3)	C7B'—N8B'	1.271 (10)
C7A—H7A	0.9500	C7B'—H7B'	0.9500
N8A—H8A1	0.83 (2)	N8B'—H8C3	0.8800
N8A—H8A2	0.83 (2)	N8B'—H8C4	0.8800
N1B—C7B	1.304 (5)	P1—F3	1.596 (2)
N1B—C6B	1.468 (7)	P1—F6	1.596 (2)
N1B—C2B	1.472 (5)	P1—F1	1.600 (2)
C2B—C3B	1.519 (6)	P1—F5	1.601 (2)
C2B—H2B1	0.9900	P1—F4	1.604 (2)
C2B—H2B2	0.9900	P1—F2	1.606 (2)
C3B—O4B	1.433 (5)	P1A—F6A	1.597 (3)
C3B—H3B1	0.9900	P1A—F1A	1.594 (2)
C3B—H3B2	0.9900	P1A—F3A	1.602 (3)
O4B—C5B	1.428 (8)	P1A—F2A	1.598 (2)
C5B—C6B	1.519 (7)	P1A—F4A	1.604 (3)
C5B—H5B1	0.9900	P1A—F5A	1.615 (3)
C5B—H5B2	0.9900	P1B—F4B	1.61 (2)
C6B—H6B1	0.9900	P1B—F3B	1.54 (2)
C6B—H6B2	0.9900	P1B—F5B	1.558 (15)
C7B—N8B	1.306 (4)	P1B—F6B	1.583 (18)
C7B—H7B	0.9500	P1B—F1B	1.594 (2)
N8B—H8B1	0.84 (4)	P1B—F2B	1.598 (2)
N8B—H8B2	0.84 (4)		
			
C7A—N1A—C2A	124.9 (2)	N1B'—C2B'—H2C4	110.5
C7A—N1A—C6A	120.6 (2)	C3B'—C2B'—H2C4	110.5
C2A—N1A—C6A	112.1 (2)	H2C3—C2B'—H2C4	108.7
N1A—C2A—C3A	108.1 (2)	O4B'—C3B'—C2B'	109.8 (9)
N1A—C2A—H2A1	110.1	O4B'—C3B'—H3C3	109.7
C3A—C2A—H2A1	110.1	C2B'—C3B'—H3C3	109.7
N1A—C2A—H2A2	110.1	O4B'—C3B'—H3C4	109.7
C3A—C2A—H2A2	110.1	C2B'—C3B'—H3C4	109.7
H2A1—C2A—H2A2	108.4	H3C3—C3B'—H3C4	108.2
O4A—C3A—C2A	110.4 (2)	C5B'—O4B'—C3B'	110.5 (9)
O4A—C3A—H3A1	109.6	O4B'—C5B'—C6B'	112.8 (7)
C2A—C3A—H3A1	109.6	O4B'—C5B'—H5C3	109.0
O4A—C3A—H3A2	109.6	C6B'—C5B'—H5C3	109.0
C2A—C3A—H3A2	109.6	O4B'—C5B'—H5C4	109.0
H3A1—C3A—H3A2	108.1	C6B'—C5B'—H5C4	109.0
C3A—O4A—C5A	112.0 (2)	H5C3—C5B'—H5C4	107.8
O4A—C5A—C6A	111.1 (2)	N1B'—C6B'—C5B'	108.7 (7)
O4A—C5A—H5A1	109.4	N1B'—C6B'—H6C3	109.9
C6A—C5A—H5A1	109.4	C5B'—C6B'—H6C3	109.9
O4A—C5A—H5A2	109.4	N1B'—C6B'—H6C4	109.9
C6A—C5A—H5A2	109.4	C5B'—C6B'—H6C4	109.9
H5A1—C5A—H5A2	108.0	H6C3—C6B'—H6C4	108.3
N1A—C6A—C5A	108.1 (2)	N8B'—C7B'—N1B'	127.6 (8)
N1A—C6A—H6A1	110.1	N8B'—C7B'—H7B'	116.2
C5A—C6A—H6A1	110.1	N1B'—C7B'—H7B'	116.2
N1A—C6A—H6A2	110.1	C7B'—N8B'—H8C3	120.0
C5A—C6A—H6A2	110.1	C7B'—N8B'—H8C4	120.0
H6A1—C6A—H6A2	108.4	H8C3—N8B'—H8C4	120.0
N8A—C7A—N1A	125.6 (2)	F3—P1—F6	90.19 (14)
N8A—C7A—H7A	117.2	F3—P1—F1	90.29 (10)
N1A—C7A—H7A	117.2	F6—P1—F1	89.73 (11)
C7A—N8A—H8A1	120.0	F3—P1—F5	179.60 (14)
C7A—N8A—H8A2	120.0	F6—P1—F5	90.11 (17)
H8A1—N8A—H8A2	120.0	F1—P1—F5	89.99 (11)
C7B—N1B—C6B	125.4 (4)	F3—P1—F4	90.14 (15)
C7B—N1B—C2B	120.4 (3)	F6—P1—F4	179.35 (13)
C6B—N1B—C2B	111.5 (4)	F1—P1—F4	89.70 (11)
N1B—C2B—C3B	108.7 (3)	F5—P1—F4	89.57 (13)
N1B—C2B—H2B1	110.0	F3—P1—F2	89.75 (10)
C3B—C2B—H2B1	110.0	F6—P1—F2	90.58 (12)
N1B—C2B—H2B2	110.0	F1—P1—F2	179.68 (14)
C3B—C2B—H2B2	110.0	F5—P1—F2	89.97 (10)
H2B1—C2B—H2B2	108.3	F4—P1—F2	89.99 (12)
O4B—C3B—C2B	111.1 (3)	F6A—P1A—F1A	91.31 (16)
O4B—C3B—H3B1	109.4	F6A—P1A—F3A	90.92 (18)
C2B—C3B—H3B1	109.4	F1A—P1A—F3A	89.64 (15)
O4B—C3B—H3B2	109.4	F6A—P1A—F2A	89.14 (15)
C2B—C3B—H3B2	109.4	F1A—P1A—F2A	179.49 (12)
H3B1—C3B—H3B2	108.0	F3A—P1A—F2A	90.60 (15)
C5B—O4B—C3B	112.2 (4)	F6A—P1A—F4A	179.36 (17)
O4B—C5B—C6B	110.7 (5)	F1A—P1A—F4A	89.15 (16)
O4B—C5B—H5B1	109.5	F3A—P1A—F4A	89.5 (2)
C6B—C5B—H5B1	109.5	F2A—P1A—F4A	90.40 (16)
O4B—C5B—H5B2	109.5	F6A—P1A—F5A	89.85 (18)
C6B—C5B—H5B2	109.5	F1A—P1A—F5A	90.47 (15)
H5B1—C5B—H5B2	108.1	F3A—P1A—F5A	179.22 (18)
N1B—C6B—C5B	108.4 (4)	F2A—P1A—F5A	89.28 (15)
N1B—C6B—H6B1	110.0	F4A—P1A—F5A	89.69 (16)
C5B—C6B—H6B1	110.0	F4B—P1B—F3B	88.9 (10)
N1B—C6B—H6B2	110.0	F4B—P1B—F5B	92.3 (9)
C5B—C6B—H6B2	110.0	F3B—P1B—F5B	178.4 (8)
H6B1—C6B—H6B2	108.4	F4B—P1B—F6B	178.9 (9)
N1B—C7B—N8B	126.4 (3)	F3B—P1B—F6B	90.2 (9)
N1B—C7B—H7B	116.8	F5B—P1B—F6B	88.6 (7)
N8B—C7B—H7B	116.8	F4B—P1B—F1B	94.9 (9)
C7B—N8B—H8B1	120.0	F3B—P1B—F1B	94.2 (8)
C7B—N8B—H8B2	120.0	F5B—P1B—F1B	84.7 (6)
H8B1—N8B—H8B2	120.0	F6B—P1B—F1B	84.6 (7)
C7B'—N1B'—C6B'	122.1 (8)	F4B—P1B—F2B	84.6 (8)
C7B'—N1B'—C2B'	124.8 (10)	F3B—P1B—F2B	85.9 (8)
C6B'—N1B'—C2B'	111.0 (8)	F5B—P1B—F2B	95.2 (6)
N1B'—C2B'—C3B'	106.2 (9)	F6B—P1B—F2B	95.9 (7)
N1B'—C2B'—H2C3	110.5	F1B—P1B—F2B	179.49 (12)
C3B'—C2B'—H2C3	110.5		
			
C7A—N1A—C2A—C3A	103.6 (3)	C7B—N1B—C6B—C5B	−102.8 (6)
C6A—N1A—C2A—C3A	−59.2 (3)	C2B—N1B—C6B—C5B	58.6 (6)
N1A—C2A—C3A—O4A	57.4 (3)	O4B—C5B—C6B—N1B	−57.7 (7)
C2A—C3A—O4A—C5A	−58.2 (3)	C6B—N1B—C7B—N8B	−8.3 (6)
C3A—O4A—C5A—C6A	57.9 (3)	C2B—N1B—C7B—N8B	−168.2 (3)
C7A—N1A—C6A—C5A	−105.2 (3)	C7B'—N1B'—C2B'—C3B'	−101.5 (12)
C2A—N1A—C6A—C5A	58.4 (3)	C6B'—N1B'—C2B'—C3B'	62.1 (13)
O4A—C5A—C6A—N1A	−56.3 (3)	N1B'—C2B'—C3B'—O4B'	−62.4 (14)
C2A—N1A—C7A—N8A	7.9 (4)	C2B'—C3B'—O4B'—C5B'	60.8 (13)
C6A—N1A—C7A—N8A	169.3 (2)	C3B'—O4B'—C5B'—C6B'	−56.5 (10)
C7B—N1B—C2B—C3B	104.8 (4)	C7B'—N1B'—C6B'—C5B'	106.4 (9)
C6B—N1B—C2B—C3B	−57.8 (4)	C2B'—N1B'—C6B'—C5B'	−57.7 (10)
N1B—C2B—C3B—O4B	55.8 (4)	O4B'—C5B'—C6B'—N1B'	54.5 (10)
C2B—C3B—O4B—C5B	−57.4 (5)	C6B'—N1B'—C7B'—N8B'	−166.6 (8)
C3B—O4B—C5B—C6B	58.3 (7)	C2B'—N1B'—C7B'—N8B'	−4.9 (14)

### Morpholinoformamidinium hexafluorophosphate (1recryst) Hydrogen-bond geometry (Å, º)

**Table:**

*D*—H···*A*	*D*—H	H···*A*	*D*···*A*	*D*—H···*A*
C2*A*—H2*A*1···F4^i^	0.99	2.36	3.115 (3)	133
C2*A*—H2*A*2···F3^ii^	0.99	2.63	3.189 (3)	116
C6*A*—H6*A*2···F4*B*	0.99	2.42	3.17 (2)	132
C7*A*—H7*A*···F2	0.95	2.53	3.043 (3)	114
N8*A*—H8*A*1···F4^i^	0.83	2.40	3.096 (3)	142
N8*A*—H8*A*1···F5*A*^iii^	0.83	2.45	3.135 (4)	140
N8*A*—H8*A*1···F5*B*^iii^	0.83	2.33	3.043 (15)	144
N8*A*—H8*A*2···O4*A*^iv^	0.83	2.04	2.864 (3)	169
N8*B*—H8*B*1···F5^v^	0.84	2.45	3.153 (4)	141
N8*B*—H8*B*2···O4*B*^vi^	0.84	2.02	2.855 (5)	169
N8*B*′—H8*C*3···F6^vii^	0.88	2.34	3.028 (8)	135
N8*B*′—H8*C*4···O4*B*′^viii^	0.88	1.97	2.839 (12)	168

Symmetry codes: (i) *x*−1/2, −*y*+1, *z*; (ii) −*x*+2, −*y*+1, *z*+1/2; (iii) *x*+1/2, −*y*+1, *z*; (iv) −*x*+3/2, *y*, *z*−1/2; (v) *x*−1/2, −*y*, *z*; (vi) −*x*+1/2, *y*, *z*−1/2; (vii) −*x*+1, −*y*, *z*+1/2; (viii) −*x*+1/2, *y*, *z*+1/2.

### Pyrrolidinoformamidinium hexafluorophosphate (2) Crystal data

**Table:**

C_5_H_11_N_2_^+^·PF_6_^−^	*F*(000) = 992
*M**_r_* = 244.13	*D*_x_ = 1.733 Mg m^−^^3^
Monoclinic, *C**c*	Cu *K*α radiation, λ = 1.54178 Å
*a* = 12.3588 (3) Å	Cell parameters from 9886 reflections
*b* = 12.7942 (3) Å	θ = 3.7–74.5°
*c* = 12.2759 (3) Å	µ = 3.28 mm^−^^1^
β = 105.400 (1)°	*T* = 130 K
*V* = 1871.38 (8) Å^3^	Block, colourless
*Z* = 8	0.21 × 0.21 × 0.11 mm

### Pyrrolidinoformamidinium hexafluorophosphate (2) Data collection

**Table:**

Bruker D8 with PHOTON III area detector diffractometer	3450 reflections with *I* > 2σ(*I*)
Radiation source: microfocus	*R*_int_ = 0.035
φ and ω shutterless scans	θ_max_ = 74.6°, θ_min_ = 5.1°
Absorption correction: multi-scan (SADABS; Krause *et al.*, 2015)	*h* = −15→14
*T*_min_ = 0.639, *T*_max_ = 0.754	*k* = −15→15
17935 measured reflections	*l* = −15→15
3467 independent reflections	

### Pyrrolidinoformamidinium hexafluorophosphate (2) Refinement

**Table:**

Refinement on *F*^2^	H atoms treated by a mixture of independent and constrained refinement
Least-squares matrix: full	*w* = 1/[σ^2^(*F*_o_^2^) + (0.085*P*)^2^ + 2.020*P*] where *P* = (*F*_o_^2^ + 2*F*_c_^2^)/3
*R*[*F*^2^ > 2σ(*F*^2^)] = 0.049	(Δ/σ)_max_ = 0.004
*wR*(*F*^2^) = 0.135	Δρ_max_ = 0.37 e Å^−^^3^
*S* = 1.07	Δρ_min_ = −0.35 e Å^−^^3^
3467 reflections	Extinction correction: SHELXL-2018/3 (Sheldrick 2015b), Fc^*^=kFc[1+0.001xFc^2^λ^3^/sin(2θ)]^-1/4^
257 parameters	Extinction coefficient: 0.0014 (4)
2 restraints	Absolute structure: Refined as an inversion twin
Hydrogen site location: inferred from neighbouring sites	Absolute structure parameter: 0.49 (4)

### Pyrrolidinoformamidinium hexafluorophosphate (2) Special details

**Table:**

Geometry. All esds (except the esd in the dihedral angle between two l.s. planes) are estimated using the full covariance matrix. The cell esds are taken into account individually in the estimation of esds in distances, angles and torsion angles; correlations between esds in cell parameters are only used when they are defined by crystal symmetry. An approximate (isotropic) treatment of cell esds is used for estimating esds involving l.s. planes.
Refinement. Refined as a 2-component inversion twin.

### Pyrrolidinoformamidinium hexafluorophosphate (2) Fractional atomic coordinates and isotropic or equivalent isotropic displacement parameters (Å^2^)

**Table:**

	*x*	*y*	*z*	*U*_iso_*/*U*_eq_	
N1	0.3206 (4)	0.7280 (3)	0.7550 (3)	0.0306 (9)	
C2	0.3205 (5)	0.8419 (4)	0.7666 (5)	0.0359 (11)	
H2A	0.291759	0.876189	0.692152	0.054*	
H2B	0.396755	0.868571	0.802754	0.054*	
C3	0.2414 (5)	0.8601 (4)	0.8420 (4)	0.0397 (11)	
H3A	0.283785	0.863514	0.922572	0.059*	
H3B	0.198412	0.925707	0.821178	0.059*	
C4	0.1646 (4)	0.7661 (4)	0.8193 (4)	0.0358 (9)	
H4A	0.105798	0.773984	0.747162	0.054*	
H4B	0.128245	0.755416	0.881246	0.054*	
C5	0.2435 (4)	0.6762 (4)	0.8132 (4)	0.0337 (10)	
H5A	0.284091	0.651192	0.889404	0.050*	
H5B	0.202882	0.617023	0.768457	0.050*	
C6	0.3770 (4)	0.6777 (4)	0.6965 (4)	0.0347 (10)	
H6	0.370295	0.603790	0.691284	0.042*	
N7	0.4439 (4)	0.7258 (3)	0.6435 (4)	0.0409 (10)	
H7A	0.4509 (6)	0.792 (4)	0.6473 (4)	0.049*	
H7B	0.480 (2)	0.690 (2)	0.606 (2)	0.049*	
N11	0.7698 (3)	0.2303 (3)	0.3159 (3)	0.0252 (7)	
C12	0.7633 (4)	0.3449 (4)	0.2985 (4)	0.0321 (10)	
H12A	0.793006	0.382648	0.370603	0.048*	
H12B	0.684995	0.367475	0.264147	0.048*	
C13	0.8362 (4)	0.3633 (4)	0.2184 (4)	0.0374 (11)	
H13A	0.805789	0.420775	0.165124	0.056*	
H13B	0.914031	0.380689	0.260727	0.056*	
C14	0.8322 (4)	0.2588 (4)	0.1552 (4)	0.0349 (9)	
H14A	0.761520	0.251673	0.094543	0.052*	
H14B	0.896670	0.251823	0.122180	0.052*	
C15	0.8383 (4)	0.1797 (4)	0.2476 (4)	0.0340 (10)	
H15A	0.805607	0.111878	0.216390	0.051*	
H15B	0.916660	0.168610	0.292739	0.051*	
C16	0.7173 (4)	0.1803 (4)	0.3774 (4)	0.0314 (10)	
H16	0.722967	0.106294	0.380722	0.038*	
N17	0.6562 (4)	0.2270 (3)	0.4358 (4)	0.0385 (9)	
H17A	0.6505 (5)	0.289 (4)	0.4342 (4)	0.046*	
H17B	0.6253 (18)	0.194 (2)	0.473 (2)	0.046*	
P1	0.52748 (8)	0.48090 (9)	0.50675 (8)	0.0281 (3)	
F1	0.5597 (3)	0.6020 (2)	0.5039 (3)	0.0491 (8)	
F2	0.4952 (3)	0.3602 (2)	0.5089 (3)	0.0473 (8)	
F3	0.5479 (6)	0.4896 (4)	0.6384 (4)	0.0758 (15)	
F4	0.6561 (3)	0.4490 (3)	0.5209 (4)	0.0666 (11)	
F5	0.5090 (5)	0.4728 (3)	0.3746 (4)	0.0730 (14)	
F6	0.4008 (3)	0.5132 (3)	0.4886 (5)	0.0678 (13)	
P2	0.55335 (8)	0.97931 (9)	0.55060 (8)	0.0294 (3)	
F1A	0.5774 (4)	0.9564 (4)	0.6830 (3)	0.0566 (9)	
F2A	0.5294 (5)	1.0049 (3)	0.4195 (3)	0.0607 (11)	
F3A	0.4242 (3)	0.9567 (3)	0.5382 (3)	0.0530 (9)	
F4A	0.5735 (4)	0.8594 (3)	0.5287 (4)	0.0611 (11)	
F5A	0.6830 (3)	1.0047 (3)	0.5650 (4)	0.0552 (10)	
F6A	0.5350 (3)	1.1002 (2)	0.5742 (3)	0.0440 (7)	

### Pyrrolidinoformamidinium hexafluorophosphate (2) Atomic displacement parameters (Å^2^)

**Table:**

	*U*^11^	*U*^22^	*U*^33^	*U*^12^	*U*^13^	*U*^23^
N1	0.0326 (19)	0.032 (2)	0.0275 (17)	0.0026 (15)	0.0078 (14)	−0.0001 (14)
C2	0.044 (3)	0.025 (2)	0.041 (2)	−0.0009 (18)	0.017 (2)	0.0006 (18)
C3	0.051 (3)	0.037 (2)	0.039 (2)	0.004 (2)	0.025 (2)	0.002 (2)
C4	0.032 (2)	0.043 (2)	0.0352 (19)	0.0038 (18)	0.0133 (16)	0.0034 (18)
C5	0.040 (3)	0.033 (2)	0.029 (2)	−0.001 (2)	0.0112 (19)	0.0001 (18)
C6	0.036 (2)	0.032 (2)	0.037 (2)	0.0017 (17)	0.0116 (19)	0.0001 (17)
N7	0.046 (2)	0.038 (2)	0.048 (2)	0.0020 (17)	0.0283 (18)	−0.0008 (17)
N11	0.0262 (17)	0.0227 (19)	0.0269 (15)	0.0001 (13)	0.0071 (13)	−0.0027 (13)
C12	0.037 (2)	0.023 (2)	0.037 (2)	−0.0015 (17)	0.013 (2)	0.0007 (17)
C13	0.043 (3)	0.027 (2)	0.043 (3)	−0.003 (2)	0.013 (2)	0.003 (2)
C14	0.035 (2)	0.040 (2)	0.0323 (19)	−0.0024 (17)	0.0131 (16)	−0.0027 (17)
C15	0.034 (2)	0.027 (2)	0.043 (3)	0.0035 (18)	0.013 (2)	−0.0013 (19)
C16	0.035 (2)	0.0262 (19)	0.032 (2)	0.0009 (17)	0.0079 (17)	0.0040 (16)
N17	0.045 (2)	0.035 (2)	0.044 (2)	0.0030 (16)	0.0280 (18)	0.0047 (16)
P1	0.0305 (6)	0.0272 (5)	0.0297 (6)	−0.0008 (4)	0.0135 (4)	−0.0002 (4)
F1	0.066 (2)	0.0324 (14)	0.0631 (19)	−0.0144 (15)	0.0411 (17)	−0.0115 (15)
F2	0.0448 (16)	0.0285 (14)	0.075 (2)	0.0023 (12)	0.0280 (15)	0.0067 (13)
F3	0.125 (5)	0.073 (3)	0.033 (2)	0.002 (3)	0.027 (2)	0.0007 (15)
F4	0.0359 (18)	0.054 (2)	0.112 (3)	−0.0018 (17)	0.0221 (19)	−0.021 (2)
F5	0.131 (4)	0.055 (2)	0.036 (2)	−0.010 (2)	0.028 (2)	−0.0084 (15)
F6	0.0331 (19)	0.0443 (19)	0.124 (4)	0.0071 (14)	0.018 (2)	−0.002 (2)
P2	0.0316 (6)	0.0291 (6)	0.0310 (6)	0.0036 (4)	0.0144 (4)	0.0005 (4)
F1A	0.066 (2)	0.068 (2)	0.0366 (17)	−0.0045 (19)	0.0160 (15)	0.0150 (16)
F2A	0.092 (3)	0.062 (2)	0.0289 (18)	−0.004 (2)	0.0164 (18)	0.0007 (14)
F3A	0.0310 (17)	0.0565 (19)	0.072 (2)	−0.0083 (15)	0.0144 (15)	−0.0127 (17)
F4A	0.089 (3)	0.0304 (15)	0.087 (3)	0.0103 (17)	0.064 (2)	−0.0006 (16)
F5A	0.0379 (19)	0.061 (2)	0.075 (3)	0.0070 (16)	0.0291 (18)	0.0190 (18)
F6A	0.0541 (18)	0.0309 (13)	0.0541 (17)	0.0011 (13)	0.0269 (13)	−0.0058 (13)

### Pyrrolidinoformamidinium hexafluorophosphate (2) Geometric parameters (Å, º)

**Table:**

N1—C6	1.296 (7)	C13—C14	1.540 (7)
N1—C5	1.490 (7)	C13—H13A	0.9900
N1—C2	1.465 (5)	C13—H13B	0.9900
C2—C3	1.531 (7)	C14—C15	1.506 (7)
C2—H2A	0.9900	C14—H14A	0.9900
C2—H2B	0.9900	C14—H14B	0.9900
C3—C4	1.512 (7)	C15—H15A	0.9900
C3—H3A	0.9900	C15—H15B	0.9900
C3—H3B	0.9900	C16—N17	1.315 (6)
C4—C5	1.523 (6)	C16—H16	0.9500
C4—H4A	0.9900	N17—H17A	0.79 (5)
C4—H4B	0.9900	N17—H17B	0.79 (5)
C5—H5A	0.9900	P1—F5	1.580 (4)
C5—H5B	0.9900	P1—F3	1.572 (4)
C6—N7	1.331 (7)	P1—F6	1.576 (4)
C6—H6	0.9500	P1—F2	1.597 (3)
N7—H7A	0.86 (5)	P1—F4	1.606 (4)
N7—H7B	0.86 (5)	P1—F1	1.602 (3)
N11—C16	1.289 (6)	P2—F3A	1.589 (3)
N11—C12	1.481 (5)	P2—F2A	1.591 (4)
N11—C15	1.489 (6)	P2—F5A	1.598 (4)
C12—C13	1.517 (7)	P2—F1A	1.600 (3)
C12—H12A	0.9900	P2—F4A	1.588 (3)
C12—H12B	0.9900	P2—F6A	1.601 (3)
			
C6—N1—C5	123.5 (4)	C15—C14—C13	102.5 (4)
C6—N1—C2	124.2 (5)	C15—C14—H14A	111.3
C5—N1—C2	112.2 (4)	C13—C14—H14A	111.3
N1—C2—C3	103.1 (4)	C15—C14—H14B	111.3
N1—C2—H2A	111.1	C13—C14—H14B	111.3
C3—C2—H2A	111.1	H14A—C14—H14B	109.2
N1—C2—H2B	111.1	N11—C15—C14	102.1 (4)
C3—C2—H2B	111.1	N11—C15—H15A	111.4
H2A—C2—H2B	109.1	C14—C15—H15A	111.4
C4—C3—C2	103.8 (4)	N11—C15—H15B	111.4
C4—C3—H3A	111.0	C14—C15—H15B	111.4
C2—C3—H3A	111.0	H15A—C15—H15B	109.2
C4—C3—H3B	111.0	N11—C16—N17	123.1 (5)
C2—C3—H3B	111.0	N11—C16—H16	118.5
H3A—C3—H3B	109.0	N17—C16—H16	118.5
C3—C4—C5	103.3 (4)	C16—N17—H17A	120.0
C3—C4—H4A	111.1	C16—N17—H17B	120.0
C5—C4—H4A	111.1	H17A—N17—H17B	120.0
C3—C4—H4B	111.1	F5—P1—F3	179.1 (4)
C5—C4—H4B	111.1	F5—P1—F6	90.3 (3)
H4A—C4—H4B	109.1	F3—P1—F6	90.5 (3)
N1—C5—C4	100.8 (4)	F5—P1—F2	89.2 (2)
N1—C5—H5A	111.6	F3—P1—F2	91.4 (2)
C4—C5—H5A	111.6	F6—P1—F2	90.73 (19)
N1—C5—H5B	111.6	F5—P1—F4	87.9 (3)
C4—C5—H5B	111.6	F3—P1—F4	91.3 (3)
H5A—C5—H5B	109.4	F6—P1—F4	178.1 (3)
N1—C6—N7	122.4 (5)	F2—P1—F4	89.8 (2)
N1—C6—H6	118.8	F5—P1—F1	90.5 (2)
N7—C6—H6	118.8	F3—P1—F1	88.9 (2)
C6—N7—H7A	120.0	F6—P1—F1	89.2 (2)
C6—N7—H7B	120.0	F2—P1—F1	179.7 (2)
H7A—N7—H7B	120.0	F4—P1—F1	90.2 (2)
C16—N11—C12	124.0 (4)	F3A—P2—F2A	91.6 (3)
C16—N11—C15	124.4 (4)	F3A—P2—F5A	178.5 (2)
C12—N11—C15	111.5 (4)	F2A—P2—F5A	88.9 (3)
N11—C12—C13	103.2 (4)	F3A—P2—F1A	88.5 (2)
N11—C12—H12A	111.1	F2A—P2—F1A	178.7 (3)
C13—C12—H12A	111.1	F5A—P2—F1A	90.8 (2)
N11—C12—H12B	111.1	F3A—P2—F4A	90.4 (2)
C13—C12—H12B	111.1	F2A—P2—F4A	91.3 (2)
H12A—C12—H12B	109.1	F5A—P2—F4A	91.0 (2)
C14—C13—C12	104.3 (4)	F1A—P2—F4A	90.0 (2)
C14—C13—H13A	110.9	F3A—P2—F6A	90.4 (2)
C12—C13—H13A	110.9	F2A—P2—F6A	89.3 (2)
C14—C13—H13B	110.9	F5A—P2—F6A	88.3 (2)
C12—C13—H13B	110.9	F1A—P2—F6A	89.4 (2)
H13A—C13—H13B	108.9	F4A—P2—F6A	179.1 (3)

### Pyrrolidinoformamidinium hexafluorophosphate (2) Hydrogen-bond geometry (Å, º)

**Table:**

*D*—H···*A*	*D*—H	H···*A*	*D*···*A*	*D*—H···*A*
N7—H7*B*···F1	0.86	2.11	2.966 (5)	177
N7—H7*A*···F3*A*	0.86	2.47	3.207 (6)	145
N7—H7*A*···F4*A*	0.86	2.51	2.945 (6)	112
C16—H16···F6^i^	0.95	2.54	3.153 (6)	122
N17—H17*A*···F2	0.79	2.51	2.935 (5)	115
N17—H17*A*···F4	0.79	2.30	3.026 (6)	152
N17—H17*B*···F6*A*^ii^	0.79	2.23	3.019 (5)	179

Symmetry codes: (i) *x*+1/2, *y*−1/2, *z*; (ii) *x*, *y*−1, *z*.
