# Supplementary figures and images for: Crystal structures of two formamidinium hexa­fluorido­phosphate salts, one with batch-dependent disorder
Source: Acta Crystallogr E Crystallogr Commun. 2024 Jan 1;80(Pt 1):88–93. doi: 10.1107/S2056989023010848 (PMC10833365; doi:10.1107/S2056989023010848)

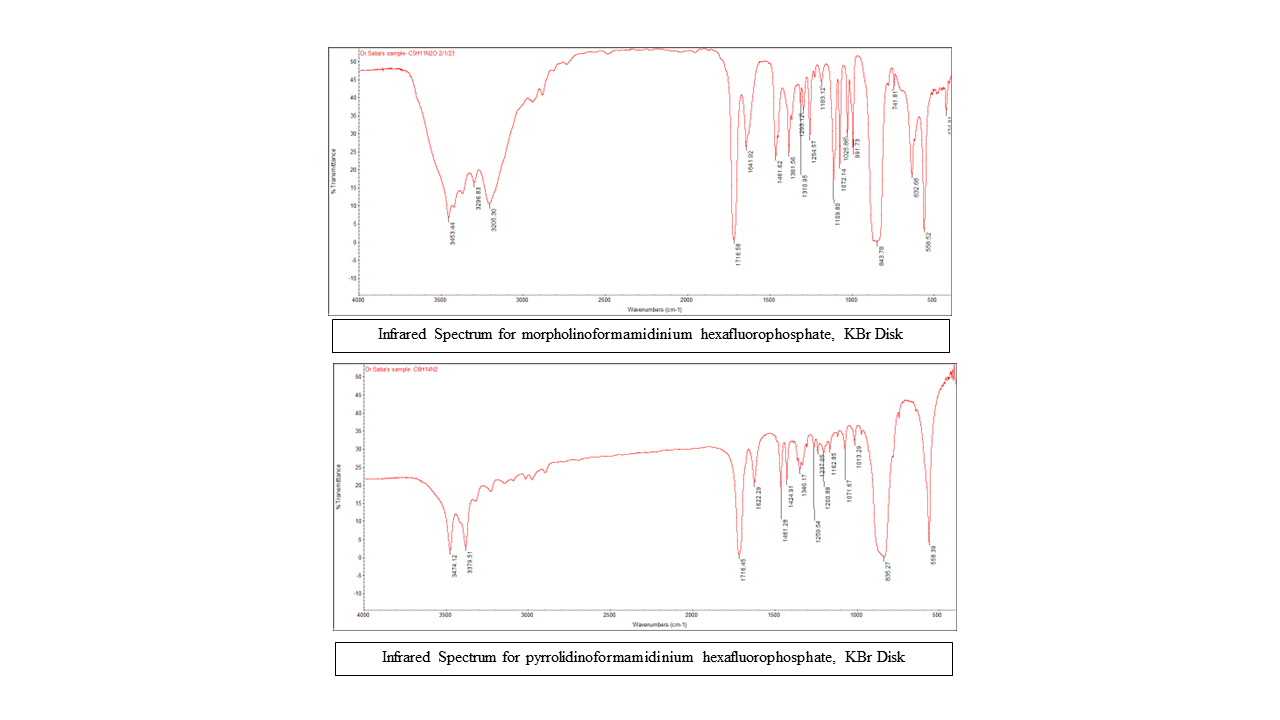

Supplement: Supplementary file 5 [file e-80-00088-sup5.tif]
